# Enhanced polarization switching characteristics of HfO_2_ ultrathin films via acceptor-donor co-doping

**DOI:** 10.1038/s41467-024-47194-8

**Published:** 2024-04-03

**Authors:** Chao Zhou, Liyang Ma, Yanpeng Feng, Chang-Yang Kuo, Yu-Chieh Ku, Cheng-En Liu, Xianlong Cheng, Jingxuan Li, Yangyang Si, Haoliang Huang, Yan Huang, Hongjian Zhao, Chun-Fu Chang, Sujit Das, Shi Liu, Zuhuang Chen

**Affiliations:** 1 School of Materials Science and Engineering, Harbin Institute of Technology, Shenzhen, 518055 China; 2 Key Laboratory for Quantum Materials of Zhejiang Province, Department of Physics, School of Science, Westlake University, Hangzhou, Zhejiang 310024 China; 3 Shenyang National Laboratory for Materials Science, Institute of Metal Research, Chinese Academy of Sciences, Wenhua Road 72, Shenyang, 110016 China; 4 Department of Electrophysics, National Yang Ming Chiao Tung University, Hsinchu, 30010 Taiwan; 5 National Synchrotron Radiation Research Center, Hsinchu, Taiwan; 6 Department of Physics, Southern University of Science and Technology, Shenzhen, 518055 China; 7 Key Laboratory of Material Simulation Methods and Software of Ministry of Education, College of Physics, Jilin University, Changchun, 130012 China; 8 Max-Planck Institute for Chemical Physics of Solids, Nöthnitzer Str. 40, 01187 Dresden, Germany; 9 Materials Research Centre, Indian Institute of Science, Bangalore, 560012 India; 10 Flexible Printed Electronics Technology Center, Harbin Institute of Technology, Shenzhen, 518055 China

**Keywords:** Ferroelectrics and multiferroics, Ferroelectrics and multiferroics

## Abstract

In the realm of ferroelectric memories, HfO_2_-based ferroelectrics stand out because of their exceptional CMOS compatibility and scalability. Nevertheless, their switchable polarization and switching speed are not on par with those of perovskite ferroelectrics. It is widely acknowledged that defects play a crucial role in stabilizing the metastable polar phase of HfO_2_. Simultaneously, defects also pin the domain walls and impede the switching process, ultimately rendering the sluggish switching of HfO_2_. Herein, we present an effective strategy involving acceptor-donor co-doping to effectively tackle this dilemma. Remarkably enhanced ferroelectricity and the fastest switching process ever reported among HfO_2_ polar devices are observed in La^3+^-Ta^5+^ co-doped HfO_2_ ultrathin films. Moreover, robust macro-electrical characteristics of co-doped films persist even at a thickness as low as 3 nm, expanding potential applications of HfO_2_ in ultrathin devices. Our systematic investigations further demonstrate that synergistic effects of uniform microstructure and smaller switching barrier introduced by co-doping ensure the enhanced ferroelectricity and shortened switching time. The co-doping strategy offers an effective avenue to control the defect state and improve the ferroelectric properties of HfO_2_ films.

## Abstract (web summary)

Ferroelectric HfO_2_ is faced with an oxygen vacancy dilemma, which favors the polar phase but harm to switching behaviors. Here, the authors propose a donor-acceptor co-doping method to enhance polarization switching characteristics of the HfO_2_ films.

## Introduction

Emerging demands for high-speed computing and high-density memory render the Von Neumann architecture less competent for future challenges. To address these issues, neuromorphic devices that mimic human synapses behaviors^1,2^ along with in-memory computing technologies^3,4^, have garnered significant attention. Ferroelectric memory devices, with stable memory state, low energy consumption, fast switching speed, and reliable endurance, emerge as promising candidates for these evolving technologies. Nevertheless, hindered by the size effect^5^ and incompatibility with standard complementary metal oxide semiconductor (CMOS) processing, the development of ferroelectric memory devices remains challenging and progresses slowly.

The situation has changed since the discovery of ferroelectricity in commercial high-*κ* HfO_2_ thin films^6^. The fluorite-structured ferroelectric thin films have attracted extensive attention in both academia and industry due to their mature preparation process and superior CMOS compatibility. Additionally, the intriguing “reverse size effect”^7^, characterized by enhanced ferroelectricity with reduced film thickness of HfO_2_, makes it a promising candidate for the next-generation, high-density, nonvolatile memory technologies, including ferroelectric random-access memory^8^, ferroelectric field-effect transistor^9^, and ferroelectric tunnel junction^10^. It is widely accepted that the structural origin of ferroelectricity in HfO_2_ films is rooted in the metastable orthorhombic phase with *Pca*2_1_ space group. Defects, such as charged oxygen vacancies, are suggested to play a crucial role in stabilizing this metastable ferroelectric phase^11–13^. Acceptor-cation doping has been identified as an effective approach to introduce charged oxygen vacancies^14^ and induce ferroelectricity in HfO_2_^15,16^. In particular, trivalent rare earth dopants have shown a pronounced effect in promoting the ferroelectric phase of HfO_2_^17^. For instance, extensive experiments have demonstrated that La-doped HfO_2_ (La:HfO_2_) films^18^ typically exhibit appreciable polarization values compared to films with various other dopants^6,19,20^. Interestingly, there are relatively few studies on the ferroelectric properties of donor doped HfO_2_ thin films. This is mainly because donor doping (e.g., Nb^5+^ doping) would suppress oxygen vacancy formation in HfO_2_ and thus favor stabilizing the non-ferroelectric monoclinic phase^21^. Despite the superb effect in triggering ferroelectricity, the role of oxygen vacancies remains a double-edged sword. While being effective in stabilizing the polar phase, oxygen vacancies can induce local structural/field inhomogeneities, thereby hindering domain wall motion^22,23^ and thus impeding the polarization response of HfO_2_-based devices^24^. As a result, the polarization switching characteristics of HfO_2_ thin films often fall behind conventional perovskite ferroelectrics like Pb(Zr,Ti)O_3_^25^. For instance, the switching time in La:HfO_2_ films remains at microsecond level^26^, nearly two orders of magnitude longer than that of Pb(Zr, Ti)O_3_, limiting the application of HfO_2_-based ferroelectrics in ultra-fast memory devices.

Understanding and enhancing the polarization switching properties of ferroelectric HfO_2_ thin films are crucial for memory applications. However, achieving both a large switchable polarization and a short switching time simultaneously in HfO_2_ films remains a challenging task. In response to the contradictory effect induced by acceptor-doping, we propose a donor (Ta^5+^)-acceptor (La^3+^) co-doping method to address this dilemma. We have identified factors affecting the switching characteristics of doped HfO_2_ films from perspectives of defective state, microstructure, lattice configuration and switching path. Benefiting from the refined defect state regulated by the co-doping strategy, the La^3+^-Ta^5+^ co-doped HfO_2_ (LT:HfO_2_) films exhibit a significantly more organized and less flawed microstructure, along with a reduced switching barrier simultaneously. A remarkable 83% increase in remanent polarization (*P*_*r*_) and a reduction in coercive fields have been achieved for the co-doped HfO_2_ samples. Moreover, robust macro-electrical characteristics of the co-doped HfO_2_ devices endure even in films as thin as 3 nm. Most notably, a substantial enhancement in switching kinetics has been achieved, manifesting as the impressively fastest switching process ever reported in HfO_2_ based ferroelectric devices.

## Results

### Structural and defective state characterizations

**Fig. 1:**
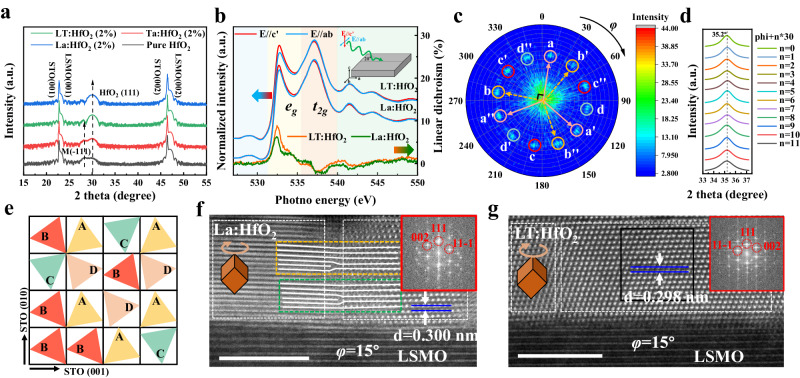
Structure characterizations and domain distribution of HfO_2_-based films. **a** XRD *θ*−2*θ* patterns of the films. **b** XAS and XLD at O-*K* edge of La^3+^ doped and LT co-doped samples. The shaded background regions with different colors represent different crystal fields, and the inset shows a schematic of the experimental configuration for the spectroscopy studies. **c** The pole figure of {002} planes observed along [111] direction of the LT:HfO_2_ film. The colored cycles in the pole figure represent 4 domain variants marked as A, B, C and D types, where the A type domain includes a (002), a’ (020) and a” (200) planes (Likewise, B type: b (002), b’ (020) and b” (200) planes; C type: c (002), c’ (020) and c” (200) planes; D type: d (002), d’ (020) and d” (200) planes). **d** 2*θ* scans of 12 diffraction points in the{002}-plane pole figure. The displayed *θ*−2*θ* data were processed with Lorentz fitting. **e** An example of HfO_2_ (111) domain distribution on the (001) plane of 4 **×** 4 STO supercells. STEM images observed along *φ* = 15° (zone axis STO [001] defined as *φ* = 0°) of (**f**) the La:HfO_2_ film and (**g**) the LT:HfO_2_ film. The rhombohedrons inset indicate the domains within HfO_2_ films with different in-plane rotation angles. The white solid lines enclosed with green and yellow dotted lines in **f** describe the dislocations.

HfO_2_-based films with thicknesses ranging from 3 to 10 nm were deposited on (001)-oriented La_0.67_Sr_0.33_MnO_3_ (LSMO)-buffered SrTiO_3_ (STO) substrates by pulsed-laser deposition (Methods and Supplementary Fig. S1a). Figure 1a displays the representative XRD *θ*−2*θ* pattern of 6 nm-thick HfO_2_, 2% La:HfO_2_, 2% Ta-doped HfO_2_ (Ta:HfO_2_), and 2% LT:HfO_2_ films. Besides the (00 *l*) reflections of the STO substrates and LSMO electrodes, peaks locating at ~30° in all films correspond to the (111)_O_ reflection of ferroelectric orthorhombic phase of HfO_2_. While the additional peak appearing around 28° in HfO_2_ and Ta:HfO_2_ films can be assigned to ($\bar{1}11$)_m_ plane of the paraelectric monoclinic phase. Remarkably, clear Laue oscillations near the HfO_2_ (111)_o_ diffraction peak is observed in the co-doped LT:HfO_2_ film, indicating the high crystalline quality and smooth interfaces. The high crystalline quality of the LT:HfO_2_ film is further supported by X-ray rocking curve studies (Supplementary Fig. S1b). The XRD studies clearly demonstrated that La:HfO_2_ and LT:HfO_2_ films have a higher fraction of ferroelectric phase than HfO_2_ and Ta:HfO_2_ films. Acceptor La^3+^ doping effectively stabilizes the metastable polar phase, consistent with previous reports^15,27^. In contrast, donor Ta^5+^ doping doesn’t seem to be an effective polar structure stabilizer, as the low energy monoclinic phase is more prone to exist. All told, LT co-doping inherits the merits of La^3+^ doping while overcoming the weakness of Ta^5+^ doping.

Furthermore, linearly polarized X-ray absorption spectroscopy (XAS) was employed to investigate the electronic structure of the films. O-*K* edge XAS reflects an unoccupied O 2*p* electronic density of states with an O 1 *s* core hole. In transition metal oxides, the O 2*p* states are significantly hybridized with the orbital states of the metal ions through the metal-oxygen orbital hybridization. In Fig. 1b, the O *K*-edge XAS of the films is depicted. The spectral weight within the pre-edge region is primarily determined by the number of holes in the O 2*p* states, projected onto the ligand orbitals^28^. These orbitals exhibit precise symmetry bonding with the Hf 5*d* orbitals. As demonstrated in Fig. 1b, the spectral weight near the pre-edge region for both La:HfO_2_ and LT:HfO_2_ films can be divided into two distinct manifolds, namely *t*_2*g*_ and *e*_*g*_, due to the crystal field splitting effect. Furthermore, the X-ray Linear Dichroism (XLD, Fig. 1b) is observed as the spectral weight difference of the XAS measurements taken with the polarization vector **E** of the incoming X-rays near parallel (**E**//c’) and perpendicular (**E**//ab) to the surface normal of the film. The observation of XLD unveils an anisotropy between the out-of-plane Hf 5*d*-O 2*p* bonding and the in-plane Hf 5*d*-O 2*p* bonding. This directly indicates the presence of a polar rhombic pyramidal distortion in both the La:HfO_2_ and LT:HfO_2_ films^7^, but differs in size. Notably, the dichroism signal of the co-doped LT:HfO_2_ film exhibits a more pronounced characteristic compared to that of the La^3+^ doped sample. This implies an amplified oxygen polyhedral distortion, indicating enhanced polarization in the co-doped film.

To gain a deeper understanding of the polar phase emerging in ferroelectric LT:HfO_2_ films, XRD pole figure measurement was carried out. Interestingly, as shown in Fig. 1c and Supplementary Fig. S2b, twelve diffraction spots appeared at *χ* ≈ 56° and 71°, respectively for {002} and {111} planes. As indicated by the colored circles in Fig. 1c and Supplementary Fig. S2b, there are four domain variants (marked as A, B, C and D) in LT:HfO_2_ films grown on LSMO buffered STO (001) substrates and each domain variant is rotated 90° in-plane with respect to each other. This rotation is attributed to the four-fold symmetry of (001)-oriented cubic substrates. However, as illustrated by the STEM results in Fig. 1f, g and Supplementary Fig. S3, domains do not strictly align by rotating 90° in sequence. As schematically shown in Fig. 1e, the four domain variants rotate randomly in plane, leading to various configurations such as ABCD, ABBB, ADAC, ACBD, BCDA, AABB, BBBB, and so on. Additionally, 2*θ* scans were conducted for the diffraction points observed in pole figures. As displayed in Fig. 1d and Supplementary Fig. S2c, the obtained results indicate a rhombohedral distortion of the polar phase. Considering the orthorhombic characteristic of freestanding HfO_2_ ferroelectric films^29^, the polar phase appearing in the HfO_2_/LSMO/STO (001) system can be defined as a orthorhombic phase with a small rhombohedral distortion.

Cross-sectional high-angle annular dark-field (HAADF) STEM images offer a detailed insight into the microstructure of doped HfO_2_ films. As seen in Fig. 1f, g, various domain variants are visible, displaying fringes and lattice forms in localized regions. The observed results are consistent with pole figures, indicating the distribution of domains with different in-plane rotation angles. As shown in Fig. 1f and Supplementary Fig. S4, numerous dislocations are present within domains and at domain boundaries in the La:HfO_2_ sample. Moreover, when examining La:HfO_2_ films from a larger area scale perspective (Supplementary Fig. S3e), additional variations like domain tilt and domain misalignment become apparent. In contrast, the situation in the LT:HfO_2_ is considerably better than that of the La:HfO_2_ films. As illustrated in Fig. 1g and Supplementary Fig. S3a, coherent domain boundaries and a uniform 0.298 nm *d*-spacing of (111) planes are evident in the LT:HfO_2_ sample. Moreover, the LT:HfO_2_ films exhibit a distinctly lower dislocation density compared to that of the La:HfO_2_ films, as shown in Supplementary Figs. S4 and S5. Considering the identical growth condition of these doped HfO_2_ films, the increased occurrence of dislocations in La:HfO_2_ films is likely associated with a higher concentration of oxygen vacancies, which usually promotes the nucleation of dislocations^30,31^. In a word, the microstructure of LT:HfO_2_ films is highly ordered, and dislocations are rare in comparison to La:HfO_2_.

**Fig. 2:**
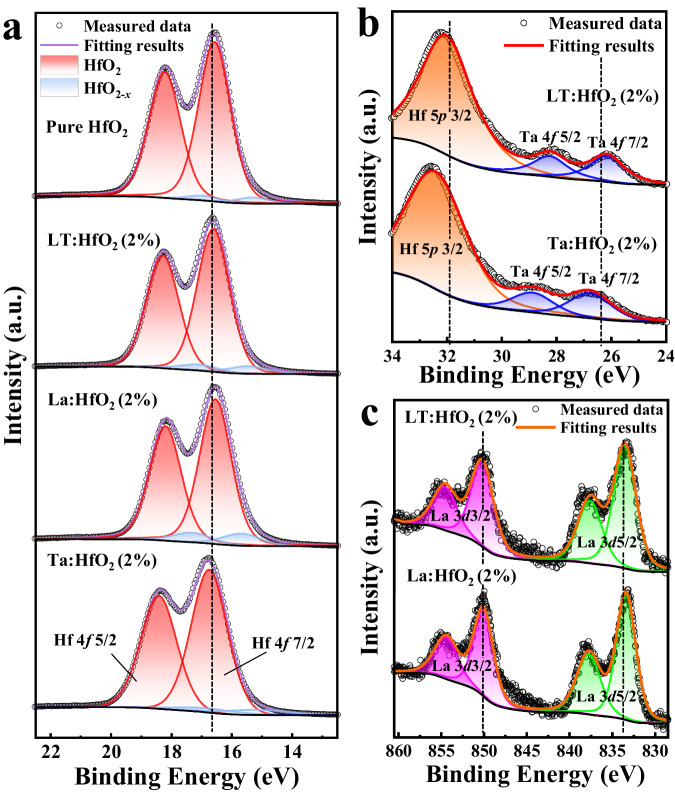
XPS of the 6-nm-thick HfO_2_ films with different doping conditions. **a** Hf 4 *f* spectra comparing pure HfO_2_ and HfO_2_-based films with different doping states. **b** Ta 4 *f* and Hf 5*p* spectra of LT:HfO_2_ and Ta:HfO_2_ films. **c** La 3*d* spectra of La:HfO_2_ and LT:HfO_2_ films.

To investigate the doping effect on the valence and defect states, X-ray photoelectron spectroscopy (XPS) studies were conducted (Fig. 2). The core level of Hf 4*f*_7/2_ from the undoped HfO_2_ film is located at 16.60 eV. Interestingly, the acceptor La^3+^ doped and donor Ta^5+^ doped films exhibit the contrary chemical shifts of the Hf 4 *f* core levels. In comparison to undoped HfO_2_, the Hf 4*f*_7/2_ binding energy of the Ta:HfO_2_ shifts to a higher region (16.75 eV), while that of the La:HfO_2_ moves to a slightly lower position (16.50 eV). The O 1 *s* spectra of La:HfO_2_ and Ta:HfO_2_ films present the same shift trend (Supplementary Fig. S6). Similar phenomena have been observed in oxides that undergo reduction or oxidation process^32–34^. Meanwhile, different chemical shifts observed in Ta 4 *f* and La 3*d* (Fig. 2b, c) peaks rule out the influence of work function changes caused by doping on the binding energy. According to the defect equation of La^3+^ doping (${{{{{\rm{La}}}}}}_{{{{{\rm{2}}}}}}{{{{{\rm{O}}}}}}_{{{{{\rm{3}}}}}}\mathop{\longrightarrow }\limits^{2{{{{\rm{Hf}}}}}{{{{{\rm{O}}}}}}_{2}}2{{{{{\rm{La}}}}}}_{{{{{\rm{Hf}}}}}}^{{\prime} }+{{{{{\rm{V}}}}}}_{{{{{\rm{O}}}}}}^{..}+3{{{{{\rm{O}}}}}}_{{{{{\rm{O}}}}}}$), oxygen vacancies are introduced to maintain charge neutrality, resulting in a reduced environment. Similarly, the reverse effect occurs when Ta^5+^ acts as a dopant in HfO_2_. In the case of the Ta:HfO_2_ film, the extra oxygen could mitigate the effect of oxygen vacancies. Consequently, the co-doping of La^3+^ and Ta^5+^ neutralizes the influence of single component doping, and the Hf 4 *f* signals virtually return to almost the same position (16.62 eV) as that of undoped HfO_2_. Moreover, the presence of Hf 4 *f* signals from HfO_2-*x*_ suboxide serves as direct evidence of the defect condition after doping. As shown, the suboxide signal is more intense in La^3+^ doped HfO_2_ due to the introduction of more oxygen vacancies; yet, Ta^5+^ doping has the opposite effect, leading to the nearly-dismissed Hf 4 *f* signature of suboxide. Thus, the co-doping method provides a simple but effective way to regulate the oxygen content and oxygen vacancies in doped-HfO_2_ films.

### Ferroelectric properties

**Fig. 3:**
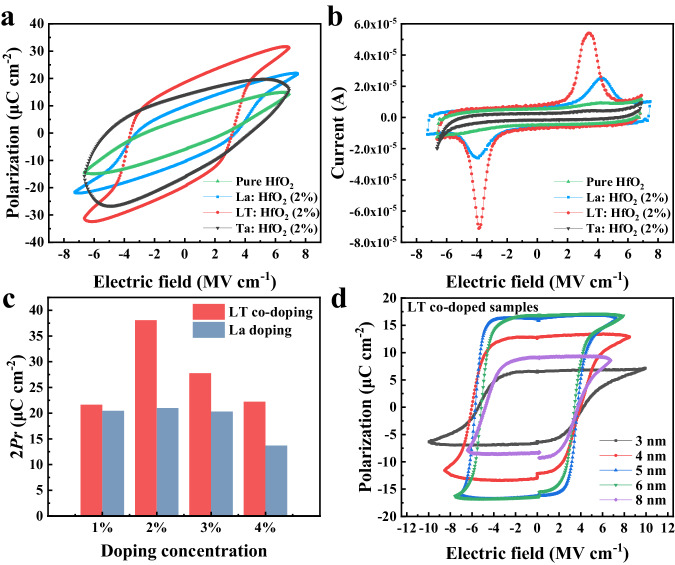
Electric measurements of doped HfO_2_ films. **a** P-E loops and (**b**) corresponding I-V curves of 6-nm-thick HfO_2_ films with different doping conditions. **c** Comparison of remnant polarization values of 6-nm-thick La^3+^ doped and LT co-doped HfO_2_ films with different doping concentrations. **d** PUND curves of LT co-doped HfO_2_ films with different thicknesses.

The electrical performances of HfO_2_ films with different doping types were further characterized. Polarization-electric field loops and corresponding I-V results of 6-nm-thick films with different doping types are depicted in Fig. 3a, b. The pure HfO_2_ film exhibits weak ferroelectricity, evident from the faint current peaks during polarization switching. The situation does not improve for the Ta^5+^ doped film; in fact, the leakage current even increases, as shown in Supplementary Figs. S7h and S8c. Consistent with XRD results, substantial improvement of ferroelectricity is achieved in both La:HfO_2_ and LT:HfO_2_ films. However, the polarization value of La:HfO_2_ is lower, and its switching current peaks appear blunter compared to those of LT:HfO_2_. This can be ascribed to a higher defect density, including oxygen vacancies, dislocations, and microstructure distortions^35–37^, which hinder the domain wall motion and consequently result in the “blunt” switching current peaks in La:HfO_2_ films. In other words, the speed of polarization reversal cannot keep up with the change of the external electric field, leading to relatively lower polarizations. In contrast, the co-doped film exhibits a significantly enhanced polarization with sharper switching current peaks, which benefits from the uniform microstructure with fewer defects. The 2% LT:HfO_2_ film has a 2*P*_*r*_ of 38 μC cm^−2^, which is 83% higher than that of the La:HfO_2_ film, consistent with aforementioned XLD results. Moreover, the coercive field *E*_*C*_ of the co-doped film decreases. Furthermore, a significant enhancement in remnant polarization values is observed in all co-doped samples (Fig. 3c and Supplementary Fig. S7c, S7d), regardless of the doping levels. LT co-doped samples with varying film thicknesses are further characterized (Fig. 3d, Supplementary Fig. S7g and S7i). Robust and reliable ferroelectricity is identified in the LT:HfO_2_ films even down to a thickness of just 3 nm. This result signifies the high film quality and less defective microstructure achieved through the co-doping method, which guarantees less resistive switching current in the ultrathin wide bandgap material (4.41 eV for *Pca*2_1_ HfO_2_^38^). This ensures the macroscopic ferroelectricity of the 3 nm thick sample. Notably, to our best knowledge, this is one of the thinnest films whose reliable macroscopic ferroelectricity can be directly obtained^39,40^. Meanwhile, the leakage current and endurance behaviors of these LT:HfO_2_ thin films remain comparable to those of La:HfO_2_ films (Supplementary Figs. S7h, S7i, S8). Altogether, these findings highlight that the co-doping strategy not only effectively enhances the ferroelectric performances of HfO_2_ films, but also elevates the quality of ultrathin films, which expand the range of possibilities for ultrathin devices.

### Switching dynamics characterizations

**Fig. 4:**
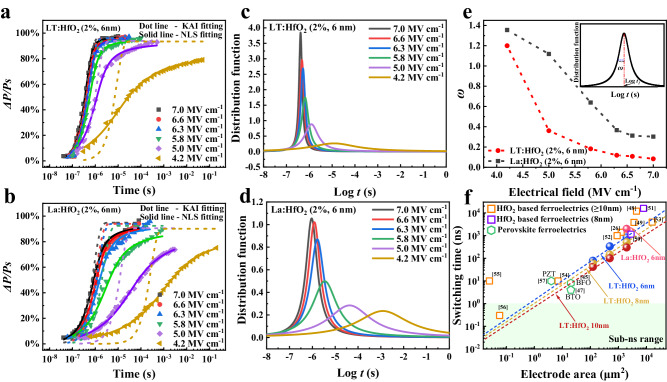
Switching dynamics characterizations of La:HfO_2_ and LT:HfO_2_ films. Switchable polarization measured with different write amplitudes and duration time of the 6-nm-thick (**a**) LT: HfO_2_ and (**b**) La: HfO_2_ films. The dotted lines and solid lines in those pictures represent the KAI and the NLS model fitting results. Lorentzian distribution functions of 6 nm (**c**) LT:HfO_2_ and (**d**) La:HfO_2_ films. **e** The changing tendency of *ω* as a function of applied electric fields. **f** Demonstration of the switching time of La:HfO_2_ and LT:HfO_2_ capacitors with different thicknesses and electrode areas and comparison with the switching time of other ferroelectric devices. Dashed lines are the linear fitting results of our devices and the shaded region represents the sub-nano-second switching time region.

As depicted above, the presence of “sharper” switching current peaks and reduced *E*_*C*_ collectively indicate the faster switching dynamics observed in the co-doped films. Therefore, the switching kinetics of doped-HfO_2_ films are investigated via pulse switching measurements using a designed pulse train sequence illustrated in Supplementary Fig. S9. Figure 4a, b displays the switched polarization fraction as a function of time at varying external fields for LT:HfO_2_ and La:HfO_2_ films, respectively, tested on top electrodes with a diameter of 50 μm (*d* = 50 μm). Overall, the co-doped film exhibits a notably faster switching behavior than the La:HfO_2_ films. In specific, a complete switching takes about 2000 ns for the La:HfO_2_ device, whereas it just spends less than 700 ns for the co-doped sample.

Polarization switching in ferroelectrics involves the nucleation of the reversed domain followed by the domain growth. Two prevalent models, the nucleation limited switching (NLS) model^41,42^ and Kolmogorov-Avrami-Ishibashi (KAI) model^43,44^ are commonly used to interpret the switching kinetics of ferroelectrics. The NLS model, which assumes that the polarization switching is limited by the domain nucleation, is often employed to explain inhomogeneous systems such as ferroelectric ceramics and polycrystalline films^42^. On the other hand, the KAI model, based on the assumption that the switching is dominated by the domain growth, has been successfully used to describe domain switching kinetics in homogeneous system, such as single crystals and epitaxial films. Both models can be interpreted by the following equation:

1 $$\Delta {{P}}\left({t}\right)=2{{P}}_{{S}}{\int }_{-\infty }^{+\infty }\left[1-\exp \left\{-{\left(\frac{{t}}{{{t}}_{0}}\right)}^{{n}}\right\}\right]{F}(\log {{t}}_{0}){{{{{\rm{d}}}}}}(\log {{t}}_{0})$$

Where *P*_*S*_ is the spontaneous polarization, F(log*t*_0_) is a probability distribution function of the characteristic switching time *t*_0_, and *n* is the effective dimension of domain growth. The F(log*t*_0_) is specific for different models. For the NLS model, influenced by defects such as oxygen vacancies, grain boundaries, and impurities, the nucleation of reversed domains does not take concerted manner and thus the distribution function is a Lorentzian function:

2 $${{{{{\rm{F}}}}}}(\log {t}_{0})=\frac{A}{\pi }\left[\frac{\omega }{{(\log {t}_{0}-\log {t}_{1})}^{2}+{\omega }^{2}}\right]$$

Where *A* is a normalized constant; *ω* and log*t*_1_ are the half-width at half-maximum and center of the distribution, as presented in the inset of Fig. 4e. While for the KAI model, *ω* approaches zero and the distribution function F(log*t*_0_) becomes a Dirac-delta function and Eq. (1) of this model can be written as:

3 $$\Delta {P}\left({{{{\rm{t}}}}}\right)=2{{P}}_{{{{{\rm{S}}}}}}\left[1-\exp \left\{-{\left(\frac{{t}}{{{t}}_{0}}\right)}^{{{{{\rm{n}}}}}}\right\}\right]$$

Therefore, the KAI model can be considered as a special case of the NLS model. Consequently, both the NLS model and KAI model are employed to fit the measured data. As demonstrated in Fig. 4a, b, only the NLS model yields a satisfactory fit for the switching kinetics of the La:HfO_2_ film. Conversely, the switching kinetics of the LT:HfO_2_ film can be equally well described by both the NLS and KAI models (above 5 MV cm^-1^). The Lorentzian distribution functions used to fit the switching kinetics of the LT:HfO_2_ and La:HfO_2_ films are presented in Fig. 4c, d, respectively. With increasing applied fields, log*t*_1_ decreases, and the Lorentzian distribution functions become sharper, indicating that defects, impurities, and other local potential variations of inhomogeneous systems are smoothed out under sufficiently large electric fields. Figure 4e illustrates the trend of *ω* as a function of electric fields, where the co-doped films consistently show smaller *ω* across all fields. In the case of co-doped LT:HfO_2_ films, the ω approaches zero when the electric field exceeds 5 MV cm^-1^, suggesting the validity of the KAI model. In contrast, the continuous change of ω with increasing applied electric fields suggests an NLS-type switching for La:HfO_2_ films. Concurrently, impacts of the imprint phenomenon on the switching dynamics are also investigated (Supplementary Fig. S10). A similar changing tendency of the switching model is observed in the negatively poled region, proving the validity of our experiment. The change in the switching mechanism further confirms a homogeneous and less defective microstructure achieved through the co-doping method. In addition, faster kinetics can be achieved with thicker films or when the capacitor areas are further minimized^45–47^. Thus, as highlighted in Supplementary Fig. S11, the switching dynamics become faster as electrode areas are reduced. For the 6-nm-thick co-doped LT: HfO_2_ film, a full switching completes within 78 ns as measured on a capacitor with an electrode diameter of *d* = 12.5 μm. Accordingly, the switching speed is further enhanced in thicker films, which is greatly attributed to the reduced depolarization fields for thicker samples. For example, a 10-nm-thick LT: HfO_2_ film completes its switching within 50 ns (tested on a *d* = 12.5 μm electrode). Therefore, it is predicted that the switching time of the films could be further minimized to the sub-ns range^45,47^, as summarized in Fig. 4f ^26,45,47–57^. The fit lines (dashed lines) exhibit the evolution of switching time versus capacitor area and indicate that the 10-nm-thick LT:HfO_2_ devices embody sub-ns switching with a capacitor area of ~2 μm^2^. The switching speed of LT:HfO_2_ films, as presented in this study, stands out as the top among the reported doped HfO_2_ systems by linear extrapolation and is comparable with that of perovskite-structured ferroelectrics. All told, our results unequivocally demonstrate that the co-doping strategy stands as an effective method to enhance the switching behaviors of ferroelectric HfO_2_ thin films.

### Atomistic insight into the switching performances in HfO_2_

**Fig. 5:**
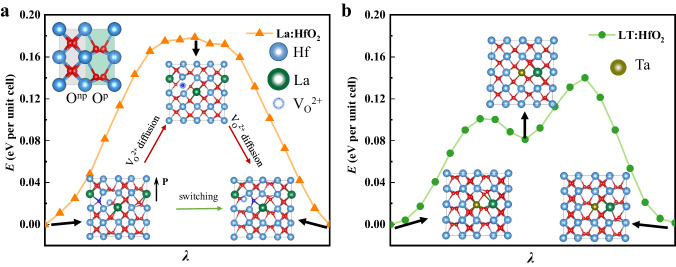
Energy profiles for polarization switching and switching paths of doped HfO_2_ in a 2×2×1 supercell. **a** The energy landscape for polar switching of the La-HfO_2_ system which contains 2 La cations and 1 oxygen vacancy. Insets show the nonpolar O^2-^(O^np^) and polar O^2-^(O^p^) in a HfO_2_ unit cell and the original, intermediate and final configurations of La-HfO_2_ during the polarization switching. **b** The switching energy profile of the co-doped HfO_2_ sample containing a La cation and a Ta cation. Insets present the switching pathway for the co-doped HfO_2_.

Density functional theory (DFT) calculations were carried out to provide atomistic insights into the structural properties of ferroelectric HfO_2_ with various doping types and to elucidate the mechanism underlying the enhanced switching properties in co-doped films. As detailed in the Supplementary Figs. S13–S15, a 2 × 2 × 1 supercell is used to model HfO_2_ systems with different doping types. For La-doped HfO_2_, we discover that the lowest-energy configuration has a three-fold coordinated (positively charged) oxygen vacancy near two La^3+^ ions substituting two Hf^4+^ ions. The presence of charged oxygen vacancy ensures the charge neutrality. In contrast, the lowest-energy configuration for LT:HfO_2_ features neighboring La^3+^ and Ta^5+^ ions, each replacing a Hf^4+^ ion. Noteworthy is that there exist multiple switching pathways in ferroelectric HfO_2_^58,59^. As shown in Supplementary Figs. S16–S18, the SI−2 switching routine is chosen as the pathway for the polarization reversal. The pathway with the lowest barrier involves concerted movements of three-fold coordinated (polar) and four-fold coordinated (nonpolar) oxygen ions against the direction of the applied electric field. As a result, the coordination number of oxygen ions changes: the initially three-fold coordinated polar oxygen ions become four-fold coordinated, whereas the four-fold coordinated nonpolar oxygen ions transit to three-fold coordination. Interestingly, we find that the polarization switching in La:HfO_2_ (Fig. 5a) is coupled to the migration of a three-fold coordinated oxygen vacancy, a process that is likely to be rather slow kinetically. The migration of the oxygen vacancy retains its three-fold coordination, as previous DFT studies have demonstrated that the charged oxygen vacancy strongly favors three-fold coordination^11^. In comparison, the switching process in LT:HfO_2_ does not involve the slow migration of vacancies. Additionally, La^3+^-Ta^5+^ co-doping lowers the energy of the intermediate state (*Pbcn*-like, Fig. 5b), thereby reducing the switching barrier. These results suggest that the co-doping strategy reduces the coercive field by mitigating the adverse impact of charged oxygen vacancies on polarization switching. At the same time, switching barriers of models with different La/Ta mole ratios and supercells (indicating different doping concentrations) are also calculated. As can be seen in Supplementary Figs. S19, S20, the La/Ta model with a 1:1 ratio facilitates the switching process than that of the La single doping, which is consistent with the experiment results.

## Discussion

In summary, we have systematically explored the effects of acceptor-donor co-doping on the microstructures, ferroelectric properties, and switching kinetics of ultrathin HfO_2_ films. Compared with La^3+^ doped films, La^3+^-Ta^5+^ co-doped HfO_2_ films exhibit a notably more uniform and less defective microstructures, changing the switching mechanism from nucleation limited switching to Kolmogorov-Avrami-Ishibashi model. First-principles calculations reveal at the atomistic level that co-doping could inhibit the generation of oxygen vacancies and reduce the polarization switching barrier. These synergistic effects of co-doping manifest in the reduction of switching barriers, leading to reduced coercive fields, enhanced polarization values, shortened characteristic switching time and faster switching speed. Furthermore, the reduced defects in co-doped HfO_2_ enable the production of high-quality films, which exhibit excellent macro-electrical performances even at a thickness of 3 nm, paving the way for advanced ultra-thin devices. The defect-engineering strategy provides an effective avenue to regulate the defect state and subsequently improve ferroelectric properties of HfO_2_-based devices.

## Methods

### Thin-film growth

HfO_2_-based films were grown on La_0.67_Sr_0.33_MnO_3_ buffered-SrTiO_3_ (001) substrates by pulsed laser deposition (Arrayed Materials RP-B) using a KrF excimer laser (*λ* = 248 nm). The thicknesses of these films were determined by the X-ray reflection (XRR) technique (Supplementary Fig. S1). The LSMO was deposited with a laser (3 HZ, 0.85 J cm^−2^) under an oxygen partial pressure of 20 Pa at the substrate temperature of 700 °C. Subsequently, the HfO_2_-based films (including the pure HfO_2_ and doped HfO_2_ samples, i.e., La:HfO_2_, Ta:HfO_2_ and LT:HfO_2_ samples) were grown at a temperature of 600 °C in a dynamic oxygen pressure of 15 Pa at a laser repetition rate of 2 Hz and a laser fluence of 1.35 J cm^−2^. Then, the heterostructures were cooled down to room temperature at the rate of 10 °C min^-1^ under an oxygen pressure of 10000 Pa. In addition, the composition of HfO_2_-based films with different doping concentration is controlled by stoichiometric PLD targets. For instance, the 2% La:HfO_2_ films are prepared using the Hf_0.98_La_0.02_O_1.99_ ceramic target, and the 2% LT:HfO_2_ films are synthesized with the Hf_0.96_La_0.02_Ta_0.02_O_2_ (i.e., the La and Ta are co-doped in a 1:1 mole ratio) target. All the ceramic targets were synthesized at 1500 °C via the solid-state reaction using HfO_2_ (99.99% purity), La_2_O_3_ (99.99% purity) and Ta_2_O_5_ (99.99% purity) powders. The top electrode Pt [ZhongNuo Advanced Material (Beijing) Technology] with a thickness of 100 nm was patterned by photolithography and deposited by magnetron sputtering (Arrayed Materials RS-M).

### Structural analysis

Structural characterization was performed by high-resolution X-ray diffraction with Cu *K*_*α*1_ radiation using a Rigaku Smartlab (9 kW) diffractometer. Two scanning modes were employed to investigate the crystal structure of HfO_2_ films. One is the radial scanning mode, in which the incident and detector move with *θ*−2*θ*, that used to analyze the orientation and lattice constants of films. The other is the pole figure mode that tilts the sample plane along *χ* direction and rotates the sample azimuthal angle *φ* with the *θ*−2*θ* position fixed. The pole figure method offers the symmetrical characteristics along certain zone axis.

### X-ray photoelectron spectroscopy (XPS) measurements

The XPS experiments were performed using an ESCALAB 250Xi instrument (Thermo Fisher) with the Al *K*_*α*_ radiation (12 kV) as the excitation source. In the XPS analysis, energy calibration was carried out by using C 1 *s* peak at 284.8 eV.

### Soft X-ray spectroscopy measurements

Soft x-ray spectroscopy measurements were achieved in the total electron yield mode (TEY) at the TLS11A and TPS45A beamline of the National Synchrotron Radiation Research Center (NSRRC) in Taiwan. Both the X-ray absorption spectroscopy (XAS) and the X-ray linear dichroism (XLD) measurements were conducted at room temperature without applied magnetic field using TEY mode. The incident angle of soft x-ray is 20° from the sample surface in XAS experiments. XLD signals were obtained from the difference of the horizontal (**E**//ab) and vertical (**E**//c’, c’ is the axis that 20° away from surface normal c) linearly polarized light absorption spectra. i.e., the polarization dependent XAS signals were guaranteed from a same sample area.

### Electron microscopy characterizations

The cross-sectional samples for STEM observations were prepared by conventional method of gluing, grinding, dimpling and ion milling. A PIPS 695 (Gatan) was used for final ion milling. At the beginning of ion milling, a voltage of 4.5 kV and an incident angle of 8° were performed to ion milled. Then, the incident angle was gradually reduced to 5°. The final voltage of ion milling was less than 0.5 kV to clean the surface damage by ion beam. Cross-sectional HAADF-STEM images and EDS mappings were acquired by Spectra 300 X-FEG aberration-corrected scanning transmission electron microscope (ThermoFisher Scientific) with double aberration (Cs) correctors and a monochromator operating at 300 kV.

### Electrical measurements

Electrical measurements were implemented via a metal-ferroelectric-metal (MFM) capacitance structure using aixacct TF3000 analyzer. The MFM capacitances were applied to electric fields with the Pt electrode connecting to the positive bias and La_0.67_Sr_0.33_MnO_3_ grounded. Diverse measurements were used to reveal macroscopic electrical performances, including the positive-up-negative-down (PUND) measurement, leakage current, capacitance-voltage (CV), switching dynamics, endurance, and polarization-electric field (P-E) loops et al. Both the PUND measurement and P-E loops were measured with a 5 kHz triangle electric pulse while the PUND measurement subtract non-switching contributions, such as dielectric and leakage current contributions.

### First-principles calculations

All first-principles density functional theory calculations were performed using Vienna Ab initio Simulation Package (VASP) with generalized gradient approximation of the Perdew-Burke-ErnZerhof (PBE) type. The polarization switching process in HfO_2_-based materials is modeled using a periodically repeated 2×2×1 supercell which contains 16 Hf and 32 O atoms. The considered polarization switching cases reported in this paper include: A. La-doped HfO_2_, in which two Hf atoms are replaced by La and an oxygen vacancy is introduced; B. La and Ta co-doping, in which two Hf atoms are replaced by a La and a Ta atom respectively, without introducing oxygen vacancy. The minimum energy paths of polarization switching in polar orthorhombic HfO_2_ under different doping conditions are determined using the variable-cell nudged elastic band (VC-NEB) technique implemented in the USPEX code with lattice constants relaxed during polarization switching. The plane-wave cutoff is set to 600 eV. We use a 2×2×4 Monkhorst-Pack k-point grid for structural optimizations and VC-NEB calculations.

In order to avoid the influence caused by excessive doping concentration, we have carried out the test calculation for the system of larger supercells (see Supplementary Information). We show the DFT minimum energy paths for polarization switching in ferroelectric hafnia with different proportions of La/Ta (1:2, 2:1 and 1:1) in 2×2×2 supercell. The plane-wave cutoff is set to 550 eV. Here we use a 2×2×2 Monkhorst-Pack k-point grid for structural optimizations and VC-NEB calculations. Additional calculations for energy barriers of SI−2 switching pathway in La:HfO_2_ and LT:HfO_2_ using 4×2×2 supercells show that the co-doped system has a much lower switching barrier than the La doped system. The plane-wave cutoff is set to 550 eV. Here we use a 1×2×2 Monkhorst-Pack k-point grid for structural optimizations and VC-NEB calculations. Other convergence conditions for VC-NEB calculations are the same: the root-mean-square forces on images smaller than 0.03 eV Å^-1^ is the halting criteria condition for NEB calculations. The variable elastic constant scheme is used, and the spring constant between the neighboring images is set in the range of 3.0 to 6.0 eV Å^-^².

### Reporting summary

Further information on research design is available in the Nature Portfolio Reporting Summary linked to this article.

### Supplementary information

Supplementary Information

Peer Review File

Lasing Reporting Summary

### Source data

Source Data

## Data Availability

The source data for Figs. 1–5 in this study are provided in the Source Data file. Source data are provided with this paper.
