# Supplementary material for: Enhanced polarization switching characteristics of HfO2 ultrathin films via acceptor-donor co-doping
Source: Nat Commun. 2024 Apr 3;15:2893. doi: 10.1038/s41467-024-47194-8 (PMC10991407; doi:10.1038/s41467-024-47194-8)
Supplement: Supplementary file 1 — Supplementary Information [file 41467_2024_47194_MOESM1_ESM.pdf]

Supplementary information

Enhanced polarization switching characteristics of HfO_2 ultrathin films *via* acceptor-donor co-doping

Chao Zhou[1], Liyang Ma[2,3], Yanpeng Feng[4], Chang-Yang Kuo[5], Yu-Chieh Ku[5], Cheng-En Liu[5] Xianlong Cheng[1], Jingxuan Li[1], Yangyang Si[1], Haoliang Huang[6], Yan Huang[1], Hongjian Zhao[7], Chun-Fu Chang[8], Sujit Das[9], Shi Liu[3,*], Zuhuang Chen[1,10,*]

[1] School of Materials Science and Engineering, Harbin Institute of Technology, Shenzhen, 518055, China

[2] Fudan University, Shanghai 200433, China

[3] Key Laboratory for Quantum Materials of Zhejiang Province, Department of Physics, School of Science, Westlake University, Hangzhou, Zhejiang 310024, China

[4] Shenyang National Laboratory for Materials Science, Institute of Metal Research, Chinese Academy of Sciences, Wenhua Road 72, Shenyang 110016, China

[5] Department of Electrophysics, National Yang Ming Chiao Tung University, Hsinchu, 30010 Taiwan

[6] Department of Physics, Southern University of Science and Technology, Shenzhen, 518055, China

[7] Key Laboratory of Material Simulation Methods and Software of Ministry of Education, College of Physics, Jilin University, Changchun, 130012, China

[8] Max-Planck Institute for Chemical Physics of Solids, Nöthnitzer Str. 40, 01187 Dresden, Germany

[9] Materials Research Centre, Indian Institute of Science, Bangalore 560012, India

[10] Flexible Printed Electronics Technology Center, Harbin Institute of Technology, Shenzhen, 518055, China

These authors contributed equally: Chao Zhou, Liyang Ma, Yanpeng Feng

*E-mail: liushi@westlake.edu.cn, zuhuang@hit.edu.cn

Structures and defect states of doped HfO_2 films

Fig. S1 displays the X-ray characterization results of HfO_2 films with different doping conditions. The thicknesses of the films are determined by the XRR method (Fig. S1a). The crystalline qualities of the films are investigated by X-ray rocking-curve studies around $(111)_o$ plane of the films in Fig. S1b. The rocking curve full width at half maxima (FWHM) of the $(111)_o$ peak was measured as small as 0.078° for LT:HfO_2, comparable to that of the STO substrate (0.02°), which suggests a high crystallinity quality of the co-doped HfO_2 film. Fig. S1c and S1d present the XRD of La:HfO_2 and LT:HfO_2 films with different doping concentrations, respectively. Peak positions of the polar phase emerging in these films do not show great differences regardless of doping concentration.

Fig. S2 shows the principle and test results of pole figures for the co-doped films. Because of the geometry similarity between the polar phase and cubic phase of HfO_2, a standard cubic structure is taken as a model to illustrate distributions of diffraction spots of {111} and {002} planes observed from the [111] direction (Fig. S2a). As can be seen from the standard model, there exists 3 spots for {111} and {002} planes, who locate at χ= 71° and 56° respectively. However, as shown in Fig. S2b, twelve diffraction spots appeared at $\chi \approx$ 71° for {111} planes. As indicated by colored circles marked in Fig. S2b (the same color suggests the refractions come from one type domain), there are 4 domain variants in the HfO_2-based films grown on LSMO buffered STO (001) substrates. Further, θ-2θ scans are performed on the 13 reflections in the {111} pole figure. As shown in Fig. S2c, identical interplanar spacings of in-plane {111} planes and an enlarged (111) plane distance hint a rhombohedral-distorted structure. This type of structure is in accordance with previous study[1]. While the elongated (111) plane distance is the result of in-plane compressive strain, Zhong *et al*[2] investigated the relaxed freestanding HfO_2 films. The 2θ of (111) plane moved to a higher angle after stripping HfO_2 films from substrates and the structure of HfO_2 turn out to be a proximate orthorhombic characteristic. Thus, more precisely, the polar phase emerging in the HfO_2/LSMO/STO (001) system can be defined as a rhombohedral distortion

orthorhombic structure.

Fig. S3 exhibits the STEM images of the doped HfO_2 films. Just as described in the main text, the microstructure of LT:HfO_2 is uniform (Fig. S3a). Whereas, domains in the La:HfO_2 display different distributions (Fig. S3e-S3i). Apart from the common in plane domain rotation, some domains exhibit a tilted posture. It is probable that some dislocations exist at the interface, whose Burgers vectors oriented out of plane, making the domain distribution tilt. In addition, although pole figures offer us an insight into the domain-variant situation within the film, the domain distribution characteristic still needs to be further investigated. As exhibited in Fig. S3b, there are two adjacent domains. According to the arrangement of Hf^{4+} ions (symbolized as yellow dots in Fig. S3b) and Fourier transform pictures (Fig. S3c-S3d), it can be judged that these two domains are rotated 180° in-plane with respect to each other. The alignment of these domains (Fig. 1f-1g, Fig. S3) proves that the 4 domain variants are not strictly aligned by rotating 90° in sequence (ABCD). We suppose that the distribution of these domains poses great impacts on ferroelectric properties of HfO_2 devices, such as wake-up phenomenon, fatigue behaviors and others. This needs to be investigated in the future work.

Furtherly, for a more intuitive exhibition of the dislocation situation, we have expanded our examination to include larger-scale images. We randomly assessed the dislocation conditions in different domains. Regions marked with 1 (I), 2 (II), 3 (III) and 4 (IV) are used to symbol different places in the La:HfO_2 film and LT:HfO_2 film. As shown in Fig. S4, to enhance clarity, the high-resolution STEM images are filtered with the Inverse Fast Fourier Transform (IFFT) method to intuitively exhibit the dislocation situations (the validity of the method is confirmed by Fig. S4a, where the defective state is more evident after the IFFT treatment). The dislocation number of the La:HfO_2 samples is significantly more than that of the co-doped samples. Furtherly, the dislocation density in each domain was calculated using the formula: $\rho = {}^{(n\times l)}\!/_{(l\times A)} = n/A$, where the n, l, A represent the number of dislocations, length of dislocation line and the area of a domain respectively. The relevant quantifiable result

(shown in Fig. S5) directly illustrates the higher dislocation numbers within the La:HfO_2 (2%) films than the LT:HfO_2 (2%) films and supports our view that oxygen vacancies favor the nucleation of dislocations.

Meanwhile, the XPS results concerning the O1*s* are shown in Fig. S6. The core level of lattice oxygen (lattice-O 1*s*) tested from the pure HfO_2 film is located at ~529.9 eV, with an additional peak located at the position ranging from 530 eV to 532 eV, representing the non-lattice oxygen (like the weakly-bonded oxygen atoms, hydroxyl and water)[3,4]. Same as Hf 4*f* spectra, La^{3+} doped and Ta^{5+} doped samples exhibit contrary trends for the change of lattice oxygen core levels. Compared with pure HfO_2, the lattice-O 1*s* binding energy of the Ta^{5+} doped sample shifts to a higher region while that of the La^{3+} doped specimen moves to a lower position. Similar phenomena have been observed in other oxides[5,6]. Hence, due to the introduction of additional oxygen vacancies, La^{3+} doping results in a decrease in the energy value of lattice-O 1*s*. Likewise, it is not difficult to interpret the reverse effect when Ta^{5+} acts as a dopant in HfO_2, where more O^{2-} are introduced and the effect of oxygen vacancies is relieved. Correspondingly, the La-Ta co-doping approach neutralizes the influence of single component doping and the lattice-O 1*s* is virtually back the same position as that of the pure HfO_2. The lattice-O 1*s* and Hf 4*f* spectra display the similar change tendency and further explain the defect states of HfO_2 based films with different doping conditions.

Ferroelectric performances of doped HfO_2 films

Ferroelectric performances and reliability behaviors of these doped-HfO_2 films are shown in Fig. S7. PUND curves and corresponding changing tendency of remnant polarizations and coercive fields (extracted from PUND curves) of these HfO_2-based films are shown in the Fig. S7a-S7b. The co-doped sample exhibits the highest polarization value (2*Pr*) of 34 μC cm^{-2}, which is much higher than that of the La doped samples, consistent with the results from dynamic P-E loop. In contrast, the pure HfO_2 and Ta:HfO_2 films present lower *Pr* values, because of the existence of a certain amount of paraelectric phase. Coercive fields of the pure HfO_2 and Ta:HfO_2 samples are likewise smaller than those of the La-doped and LT-doped devices. The La-doped

sample exhibits the largest *Ec* among all these samples. Except for the higher content of polar phase, factors like oxygen vacancy-pinned domain wall and microstructure distortion also contribute to the increased coercive field. By contrast, though the *Pr* value of the co-doped capacitor is far ahead among all the samples, the *Ec* of it is smaller than that of the La-doped one.

Meanwhile, it can be observed from Fig. S7c-S7d that no matter what doping concentrations, the polarization values of co-doped films are superior than the corresponding La^{3+} doped samples. At the same time, benefiting from the fine defect state obtained by the co-doping technique, robust P-E loops can be obtained for LT:HfO_2 (2%) films as thin as 3 nm (Fig. S7g). Concurrently, the leakage current of LT:HfO_2 (Fig. S7h) is not getting worse after Ta^{5+} doping in the La:HfO_2 system. Besides, the endurance and cycling performances of LT:HfO_2, La:HfO_2 and Ta:HfO_2 films are measured simultaneously (including the 3 nm thick LT:HfO_2 device). All samples are cycled with the 100 kHz bipolar rectangular wave and read with the 5 kHz triangle wave. Among them, the 6-nm-thick films are cycled and read under a 4 V voltage and the 3-nm-thick capacitors are cycled and read with a 2.5 V voltage. It has been found that it takes around ten cycles for the 6-nm-thick LT:HfO_2 and La:HfO_2 samples to enter the optimal state. Due to the large leakage current observed in the Ta:HfO_2 device, the *Pr* looks bigger (just as demonstrated in Fig. 3a) and the device easily went breakdown. Nevertheless, the reliability of ferroelectric HfO_2 films has been substantial improved by La^{3+} doping and LT co-doping compared with that of the Ta:HfO_2 device. Most notably, the 3-nm-thick LT:HfO_2 device exhibits exceptional reliability with an endurance over 1E8 cycles. However, it is worth noting that there still exist fatigue behaviors in the doped HfO_2 films, which need to be further improved in our future works.

We have also taken a close look at the leakage current conditions of doped-HfO_2 devices. The current density and applied electric field (*J-E*) relations of the LSMO/doped-HfO_2/Pt capacitors from both positive and negative bias in the temperature ranging from 350 K to 450 K were further studied and are depicted in Fig. S8a-S8c. Leakage currents of all films rise obviously with increasing temperatures, and

the Ta-doped HfO_2 films exhibit highest leakage current densities for all temperatures. Meanwhile, as shown in Fig. S8d-S8f, curves of $Ln(J/T^2)$ versus $E^{1/2}$ take on a linear relationship and the dynamic dielectric constant ε_{opt} of HfO_2 within reason, meaning that the leakage responses of the doped HfO_2 films obey the Schottky emission mechanism[7]. To further unveil the nature of conduction behavior, the Schottky barrier heights Φ_B are extracted from the intercepts[7] of lines in Fig. S8g-S8i. As are shown, the linear dependence further validates the Schottky emission mechanism. Both barrier heights Φ_B of LSMO/doped-HfO_2 and Pt/doped-HfO_2 interfaces of the La:HfO_2 and LT:HfO_2 are higher than those of the Ta:HfO_2 sample, directly explaining the smaller leakage current observed in the La doped and LT doped devices.

Switching dynamics measurements for different doped HfO_2 films

Fig. S9 describes the pulse sequence used for the measurement of switching dynamics. The pulse sequence can be divided into three parts, denoting as write part, read part and refresh part. Among them, the amplitude and frequency of the read pulse and the refresh pulse are 7 MV cm^{-1} and 5 kHz respectively, with a fixed delay time of 200 μs. The amplitude and duration time (τ) of the write pulse are indicated in the legend of Fig. 4, Fig. S10 and Fig. S11.

Due to the asymmetric electrode (LSMO/doped-HfO_2/Pt) configuration, a notable imprint can be observed in La:HfO_2 and LT:HfO_2 systems. The influences of imprint on the switching dynamics are also investigated. A pulse sequence with opposite directions to the designed pulses shown in Fig. S9 is employed to explore the switching behavior in the negative voltage region. As displayed in Fig. S10, the changing tendency of the switching models in the negative region shows no difference compared to that in the positive region, and the differences in switching speed between them are even more prominent during the negative switching process. The negative voltage switching process further demonstrates the superiority of the La-Ta co-doping method.

Meanwhile, pictures in Fig. S11 demonstrates the switching dynamics of LT:HfO_2 films with different thicknesses and capacitors areas. A common trend can be concluded that the switching speed gets faster as the film becomes thicker or the area of electrode

gets smaller. Fig. S11j demonstrates the switching time versus different electric fields and their relationship is fitted using Merz's law, showing as the dashed lines in the picture. Increased activation fields are observed as the thickness scaling, which clarify the effect of depolarization fields on switching barriers. Meanwhile, the La:HfO_2 sample exhibits the highest activation field, explaining the great influence of microstructure distortion and defect pining on switching. Besides, as for the faster switching speed obtained from small capacitor devices, the number of domains is reduced, which interprets a faster switching speed when using a smaller electrode.

Furthermore, *RC* time of the test circuit is determined as shown in Fig.S12 for a better assessment of switching time. we have measured the *RC* constant of La:HfO_2 and LT:HfO_2 capacitors with different electrode areas. Designed pulse was used to eliminate the polarization contribution (pre-pole and switching processes) and record the current change situation of the ferroelectric capacitor (non-switching process). As depicted in Fig. S12b, the formula $I=I_0\times e^{\frac{-t}{RC}}+b$ (I_0-maximum current, t-time; b-constant) is used to fit the curve and obtain the *RC* constants of the circuits. The *RC* constants of devices with diverse areas are plotted in Fig. S12c and they exhibit a linear relationship, consistent with the reported work[8]. Simultaneously, the *RC* time varies from 89 ns to 257 ns for electrodes with a diameter ranging from 25 μm to 100 μm (the *RC* time of La doped and LT doped samples does not show significant differences and therefore is not discussed respectively). The *RC* time of these devices is considerably shorter than the switching time and does not impact the results.

Calculations about the configurations of doped HfO_2

A. La-Vo configurations

Configurations of doped HfO_2 are taken into account as well. Firstly, an analysis was conducted for the La-Vo condition. This allowed us to determine the distribution of oxygen vacancies introduced by La doping. HfO_2 ferroelectric orthorhombic phase comprises two types of oxygen ions: 3-coordinated polar oxygen ions and 4-coordinated non-polar ones. Therefore, as revealed in previous studies[9], formation energies of oxygen vacancies at various coordination sites differ. In view of this, as

shown by positions marked in purple in Fig. S13, vacancies located at sites corresponding to the two types of oxygen ions near the substitution La atom are introduced. Calculation results show that the energy of the system is lowest when the nearest 3-coordinated oxygen ion is taken away (see Table S1). In other words, the 3-coordinated oxygen vacancy closest to the doped La atom has the lowest formation energy.

B. 2La-Vo configurations

Next, the configuration of 2La-V_O, which is considered as a more realistic charge compensation case associated with the experiment, is investigated. Based on previous results obtained from La-V_O and charge compensation, it is reasonable to assume that in the configuration of 2La-Vo, the oxygen vacancy is near the La cation, just as exhibited in Fig. S14. To determine the most stable configuration, we calculated the thermodynamic energy for numerous possible configurations, as displayed in Table S2. The La_{Hf2}-V_O-La_{Hf7} model is supposed to be the most stable configuration. On this basis, we were able to calculate the polarization switching energy barrier.

C. La-Ta co-doping configurations

In the case of co-doping, the system is electrically neutral, which leads to a lower concentration of oxygen vacancies compared to La^{3+} doping. This is advantageous as it reduces film leakage and prevents the formation of large-scale defects such as dislocations caused by oxygen vacancy pinning. Therefore, as shown in Fig. S15, we examined the configuration of hafnium oxide co-doped with La-Ta without introducing oxygen vacancies. Our analysis revealed that the thermodynamic energy of the different configurations resulting from various relative positions of the La and Ta substituted cations did not differ significantly (seen in Table S3). We identified the most stable configuration, Ta-La_{Hf14}, to be used for the subsequent polarization switching study.

Polarization switching barriers of different doped HfO_2

A. Switching pathways of pure HfO_2

Ferroelectric HfO_2 has multiple ferroelectric switching pathways, which have been described in several works[10,11]. As can be seen in Fig. S16, switching pathways in HfO_2

at the unit cell level can be divided into shift-inside (SI) and shift-across (SA)[10]. In the SI pathway, oxygen ions move between two Hf atomic planes. Specifically, the SI-1 pathway only involves the displacement of polar oxygen ions against electric field, and the transition state is tetragonal $P4_2/nmc$ phase; the SI-2 pathway has both polar oxygen atoms (O^p) and non-polar oxygen atoms (O^{np}) moving against **E**, resulting in converted $O^p \rightarrow O^{np}$ and $O^{np} \rightarrow O^p$. In contrast, polar oxygen ions move across Hf planes in the SA pathway. The switching barriers calculated with the variable-cell nudged elastic band (VCNEB) technique based on density functional theory (DFT) are 0.39, 0.22, and 0.79 eV per unit cell (u.c.) for SI-1, SI-2, and SA, respectively (Fig.S16d).

B. Switching pathways of doped HfO_2

As is shown in Fig. S16d, the intrinsic switching barriers are 0.79, 0.39 and 0.22 eV per unit cell (u.c.) for SA, SI-1 and SI-2, respectively. Clearly, the SI-2 pathway is more likely to occur. So, in this work we show the energy barrier of SI-2 switching pathway for different doping systems. We have performed additional calculations for SI-1 and SI-2 pathways in La:HfO_{2-x} and LT:HfO_2. As demonstrated in Fig. S17, the SI-2 pathway in LT:HfO_2 exhibits the lowest barrier, further corroborating our main conclusion that acceptor-donor co-doping can improve the ferroelectric switching properties.

We have reproduced the results of previous work which focus on the motion of the *Pbca*-type 180° domain wall[12,13]. Both studies reported that the "crossing" mechanism, which can be viewed as the SA pathway at the domain wall and has a lower barrier than the "non-crossing" mechanism. Our reproduced results are shown in Fig. S18a: the crossing pathway is energetically favored. Furthermore, we investigate the crossing mechanism in La:HfO_{2-x} and LT:HfO_2 using 8×1×1 supercells. As demonstrated in Fig. S18b, the co-doping slightly reduces the barrier compared to that in La:HfO_{2-x}. Importantly, we note that the barrier is ~0.35 eV for lateral motion by one unit cell in LT:HfO_2, which compares unfavorably with the homogenous SI-2 pathway with a barrier of 0.14 eV/u.c. (see Fig. S17). Unlike perovskite ferroelectrics, our results suggest that homogenous switching is kinetically favored over the motion of the *Pbca*-

type 180° domain wall.

C. The SI-2 switching pathway for doped-HfO_2

In this work we focused on the SI-2 switching pathway for its lower energy barrier. Based on the SI-2 switching path, the instability of the system with 4-coordinated oxygen vacancy results in the migration of oxygen vacancy during the polarization switching process of La^{3+} doped HfO_2. This migration is a relatively slow dynamic process, and the transition state of the migration increases the switching energy barrier. In combination with the occurrence of dislocations in La^{3+} doped systems, these factors collectively inhibit the switching kinetics of La^{3+} doped HfO_2.

At the same time, to confirm the validity and robustness of our calculations, we further carry out the simulations with more La/Ta mole ratios and perform calculations with different supercells (indicating different doping concentrations). Except for the 1:1 mole ratio of La/Ta, we have conducted additional DFT theoretical simulations for La/Ta-HfO_2 systems with two more La/Ta ratios, 1:2 and 2:1. A 2×2×2 supercell is used to compare the polarization switching energy barrier of codoping systems of different components. As shown in Fig. S19, the system with a La/Ta ratio of 1:1 has the lowest switching energy barrier. Furthermore, we have performed calculations using 4×2×2 supercells, with results shown in Fig. S20. It is evident that the co-doped system has a much lower switching barrier than the La doped system. In short, the co-doping method effectively promotes the polarization switching than the La single doping situation.

References

1. Wei, Y. *et al.* A rhombohedral ferroelectric phase in epitaxially strained $Hf_{0.5}Zr_{0.5}O_2$ thin films. *Nat. Mater.* **17**, 1095–1100 (2018).
2. Zhong, H. *et al.* Large-Scale $Hf_{0.5}Zr_{0.5}O_2$ Membranes with Robust Ferroelectricity. *Adv. Mater.* **34**, 2109889 (2022).
3. Idriss, H. On the wrong assignment of the XPS O1*s* signal at 531–532 eV attributed to oxygen vacancies in photo- and electro-catalysts for water splitting and other materials applications. *Surf. Sci.* **712**, 121894 (2021).

4. Yu, J. *et al.* Highly Stable Pt/CeO_2 Catalyst with Embedding Structure toward Water–Gas Shift Reaction. *J. Am. Chem. Soc.* **146**, 1071–1080 (2024).
5. Vovk, G., Chen, X. & Mims, C. A. In Situ XPS Studies of Perovskite Oxide Surfaces under Electrochemical Polarization. *J. Phys. Chem. B* **109**, 2445–2454 (2005).
6. Kim, B. Y. *et al.* Top Electrode Engineering for High-Performance Ferroelectric $Hf_{0.5}Zr_{0.5}O_2$ Capacitors. *Adv. Mater. Technol.* **2033146**, 2300146.
7. Pabst, G. W., Martin, L. W., Chu, Y.-H. & Ramesh, R. Leakage mechanisms in $BiFeO_3$ thin films. *Appl. Phys. Lett.* **90**, 072902 (2007).
8. Parsonnet, E. *et al.* Toward Intrinsic Ferroelectric Switching in Multiferroic $BiFeO_3$. *Phys. Rev. Lett.* **125**, 067601 (2020).
9. He, R., Wu, H., Liu, S., Liu, H. & Zhong, Z. Ferroelectric structural transition in hafnium oxide induced by charged oxygen vacancies. *Phys. Rev. B* **104**, L180102 (2021).
10. Ma, L. *et al.* Ultrahigh Oxygen Ion Mobility in Ferroelectric Hafnia. *Phys. Rev. Lett.* **131**, 256801 (2023).
11. Choe, D.-H. *et al.* Unexpectedly low barrier of ferroelectric switching in HfO_2 via topological domain walls. *Mater. Today* **50**, 8–15 (2021).
12. Silva, A. *et al.* Unraveling the ferroelectric switching mechanisms in ferroelectric pure and La doped HfO_2 epitaxial thin films. *Mater. Today Phys.* **34**, 101064 (2023).
13. Wu, Y. *et al.* Unconventional Polarization-Switching Mechanism in (Hf,Zr)O_2 Ferroelectrics and Its Implications. *Phys. Rev. Lett.* **131**, 226802 (2023).

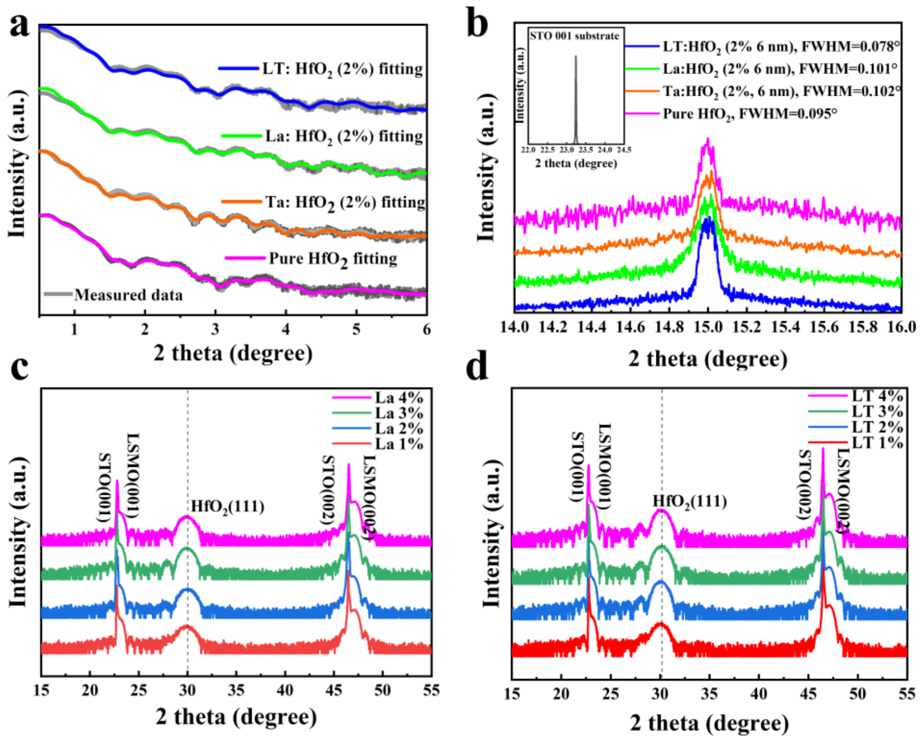

Fig. S1 | X-ray characterization results of HfO_2 films with different doping conditions. a X-ray reflections (XRR) and concerning fitting curves of the 6-nm-thick doped HfO_2 films. **b** Rocking curves of the 6-nm-thick doped HfO_2 films and inset is the rocking curve of the STO (001) substrate. The X-ray diffraction patterns of **c** La doping and **d** LT co-doping HfO_2 films with different doping concentrations.

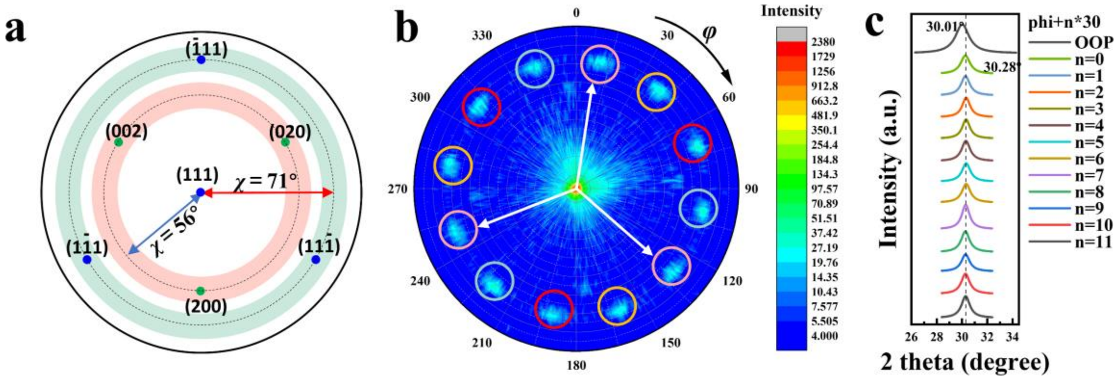

Fig. S2 | Pole figure characterization of the 6 nm LT:HfO_2 (2%) film. a Distribution of {111} planes and {002} planes observed along [111] direction in the standard Cubic structure. **b** The pole figure of {111} planes. **c** 2θ scans of 13 peaks in the {111}-plane pole figure (including the out of plane-OOP in short). The displayed θ-2θ data were processed with Lorentz fitting.

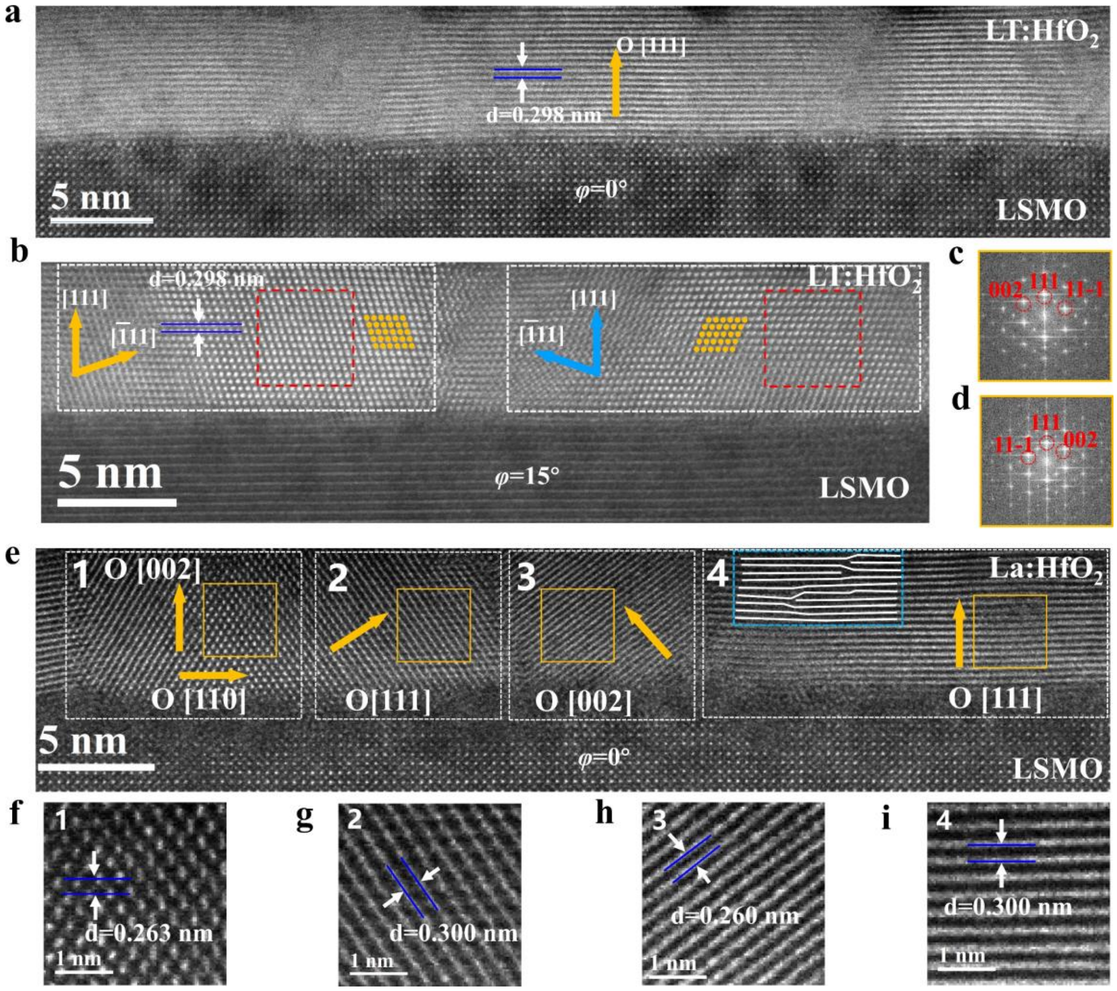

Fig. S3 | HAADF STEM images. a The LT:HfO_2 sample observed from a larger perspective scale along zone axis of STO [001] (defined as φ=0°). **b** The STEM image of the LT:HfO_2 sample observed along φ=15°. The yellow dots represent the arrangement of Hf^{4+} in the adjacent domains. **c** and **d** are the Fourier transform pictures of the left and right regions selected by the red dashed line in the **b**. **e** La:HfO_2 samples observed from a larger perspective scale along φ=0°. The blue dashed enclosed region in **e** represents the presence of dislocations. **f-i** Enlarged pictures of the 1-4 regions in the yellow enclosed regions in **e**.

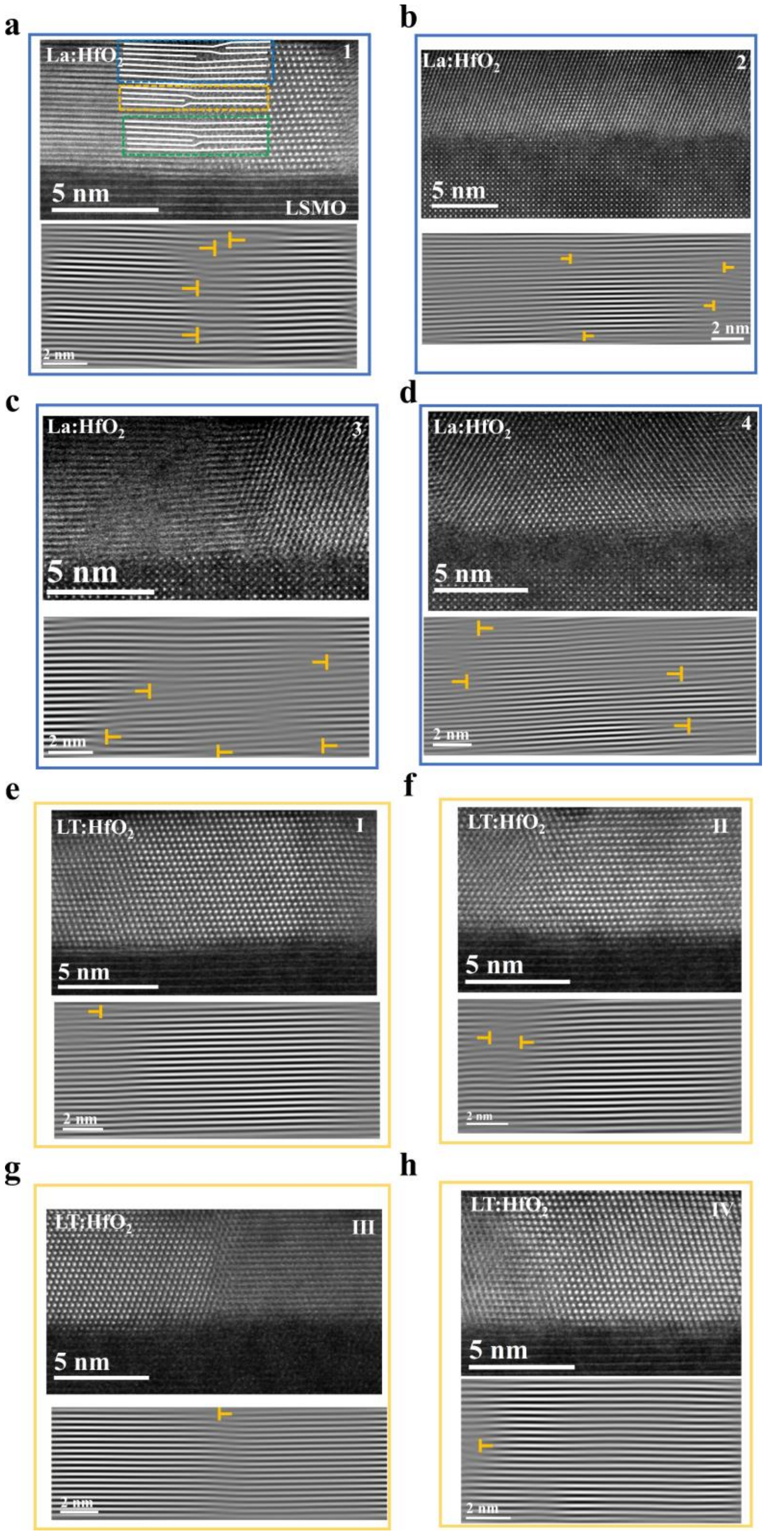

Fig. S4 | Dislocation conditions for La:HfO_2 and LT:HfO_2 films. **a-d** Region 1 to region 4 for the La:HfO_2 sample. **e-h** Region I to region IV for the LT:HfO_2 sample. The yellow marker "⊥" is used to symbol the dislocation.

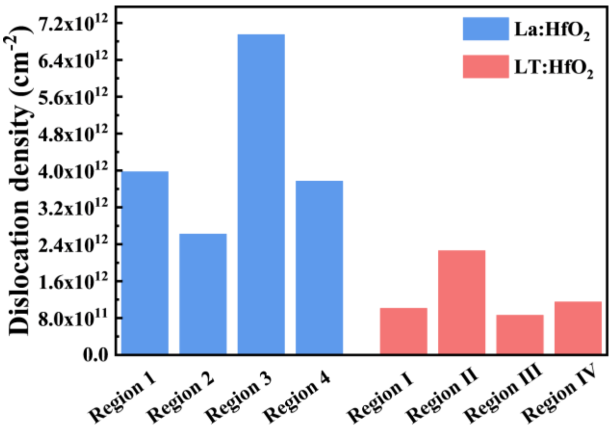

Fig. S5 | Diagram of dislocation density. The dislocation density in different domain regions within the La:HfO_2 (2%, 6 nm) and LT:HfO_2 (2%, 6 nm) films.

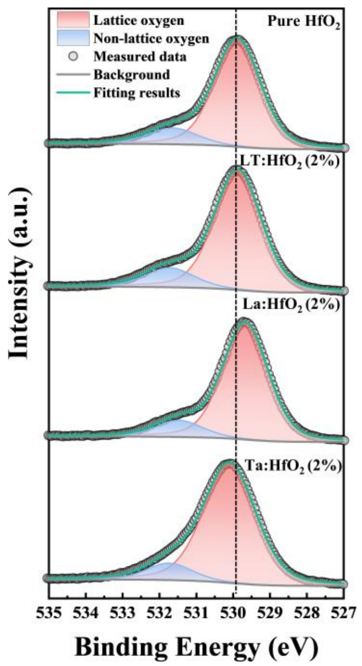

Fig. S6 | O 1*s* spectra characterization. The O 1*s* spectra of the 6-nm-thick pure HfO_2 and HfO_2-based films with different doping states.

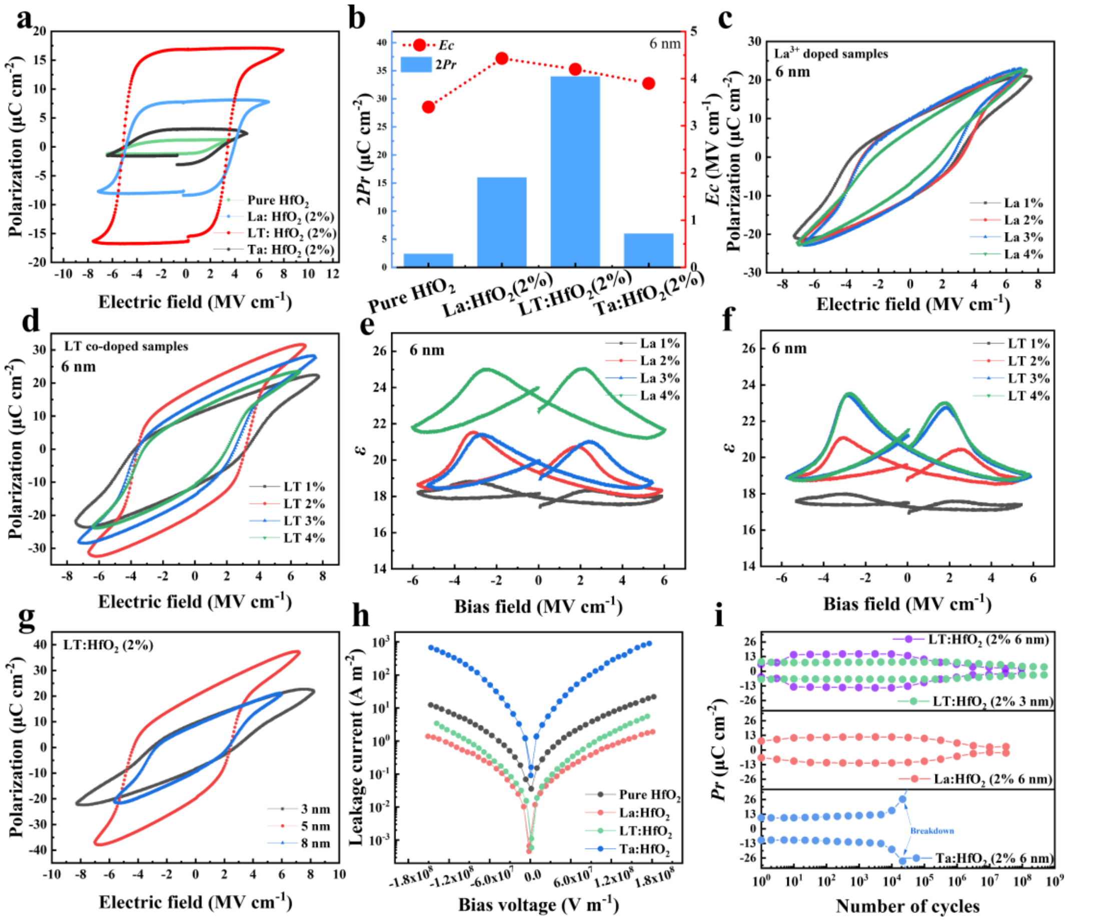

Fig. S7 | Ferroelectric performances and reliability behaviors of HfO_2 films. **a** PUND curves of HfO_2-based capacitors with different doping conditions. **b** The changing tendency of remnant polarizations and coercive fields (extracted from PUND curves) along with different doping situations. P-E loops of **c** the 6-nm-thick La^{3+} doped and **d** 6-nm-thick LT co-doped samples with different doping concentrations and their ε-V curves are displayed in **e** and **f** respectively. **g** P-E loops of the LT:HfO_2 (2%) films with different thicknesses. **h** Leakage currents of 6-nm-thick 2% doped-HfO_2 samples with different doping types. **i** Endurance measurements of doped-HfO_2 devices with different thickness and doping conditions.

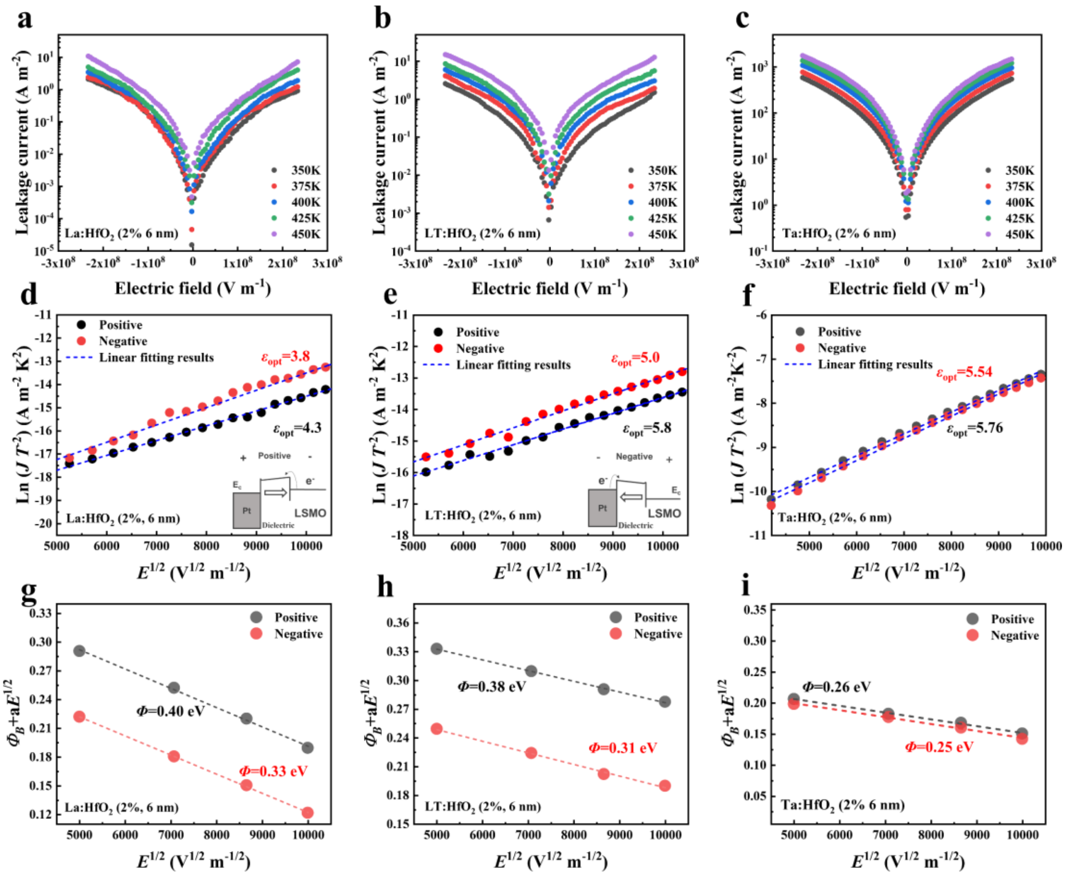

Fig. S8 | The leakage currents and conduction mechanism analysis of doped-HfO2 films. Leakage currents at different temperatures for the **a** La:HfO_2, **b** LT:HfO_2 and **c** Ta:HfO_2 devices. The Schottky emission mechanism fitting results for the **d** La:HfO_2, **e** LT:HfO_2 and **f** Ta:HfO_2 devices. The inset diagrams in **d** and **e** illustrate the positive and negative concepts. Barriers from the positive and negative bias for the **g** La:HfO_2, **h** LT:HfO_2 and **i** Ta:HfO_2 devices.

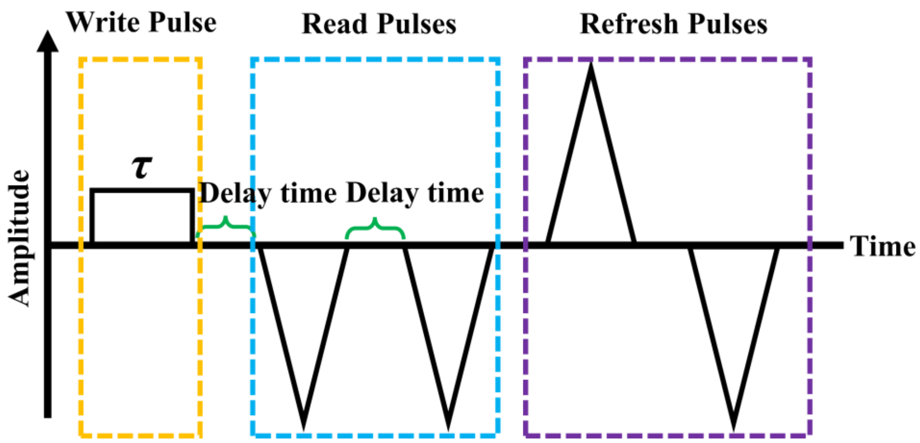

Fig. S9 | Pulse illustration. Pulse sequence used for switching dynamic test.

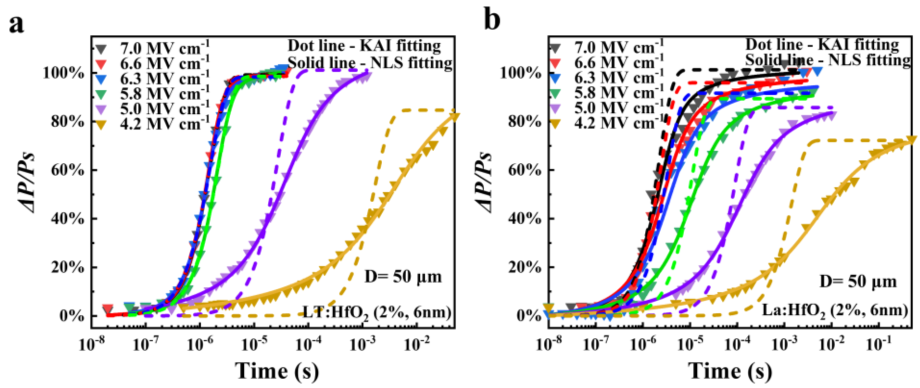

Fig. S10 | The impact of the imprint on the switching dynamics. The switching dynamics of the **a** LT:HfO_2 (2%, 6 nm) and **b** La:HfO_2 (2%, 6 nm) samples testing on D=50 μm electrodes. The testing pulse sequence is the signal equipped with the opposite dirctions to the pulse sequence in the Fig. S9.

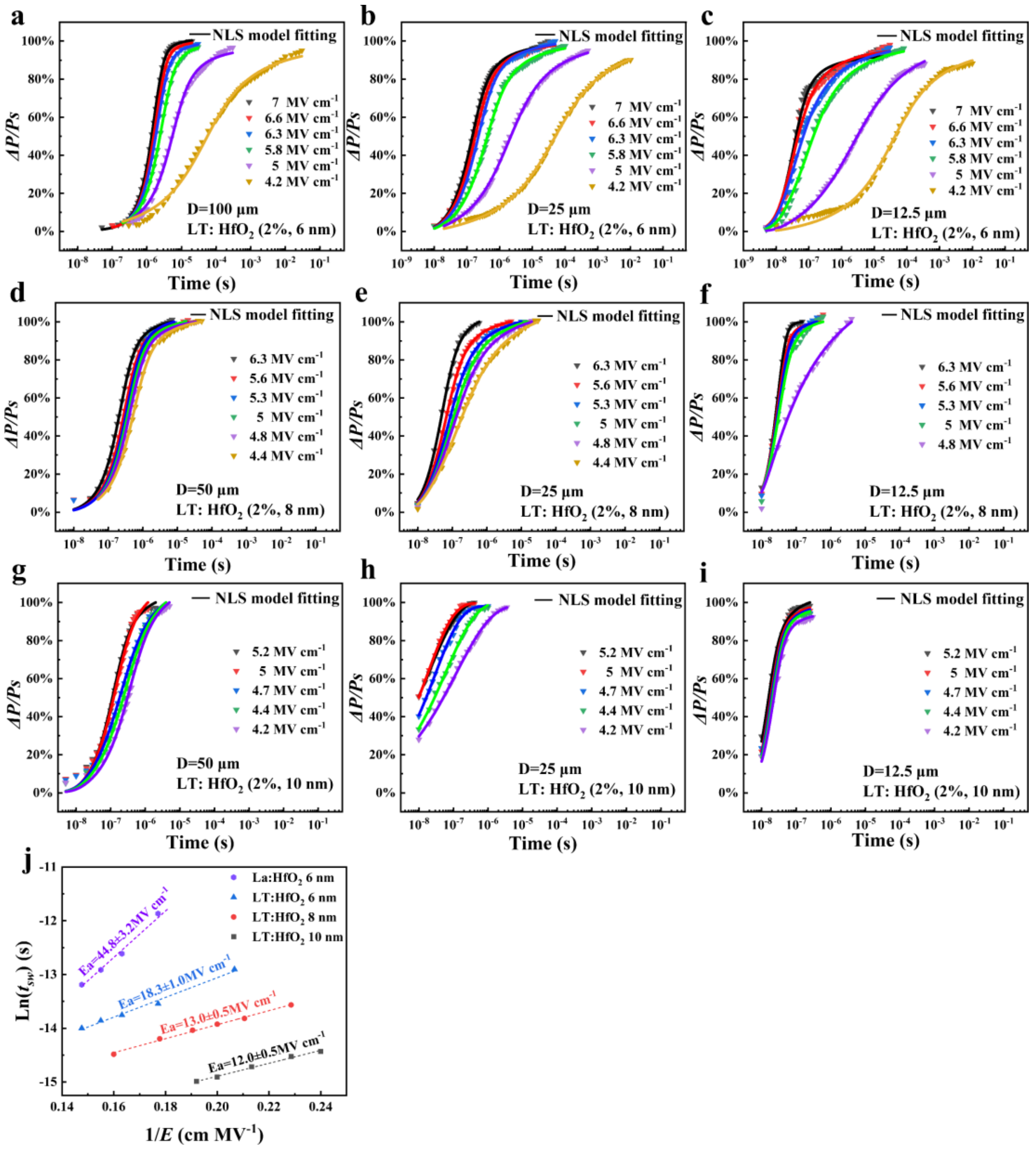

Fig. S11 | Switching dynamics of LT:HfO2 films with different tested electrode areas and film thicknesses. The switchable polarization versus duration time under different applied electric fields of **a-c** 6 nm LT:HfO_2, **d-f** 8 nm LT:HfO_2 and **g-i** 10 nm LT:HfO_2 films tested with different electrode sizes. **j** Activation fields of La:HfO_2 and LT:HfO_2 films with different thicknesses. The dashed lines in **j** are the fitting results using Merz's law.

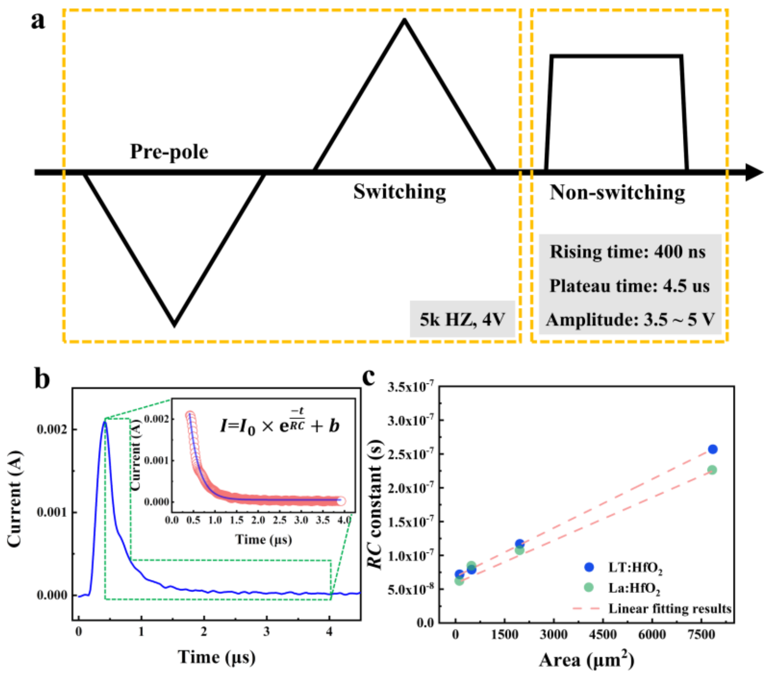

Fig. S12 | *RC* constant of the La:HfO_2 (2%, 6 nm) and LT:HfO_2 (2%, 6 nm) capacitors. a The pulse sequence used to measure the *RC* constant. **b** The measured current signal of the La:HfO_2 (2%, 6 nm) capacitor with an area of 7850 μm^2. Inset is the fitting result to extract the *RC* time. **c** The *RC* time of the La:HfO_2 (2%, 6 nm) and LT:HfO_2 (2%, 6 nm) capacitors with different areas.

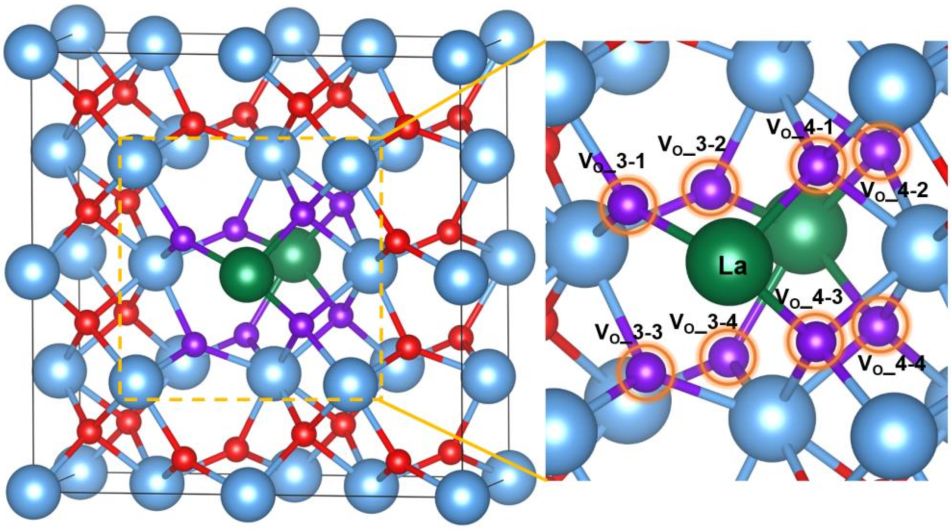

Fig. S13 | Schematic of the location of an oxygen vacancy at different coordination sites. The enlarged picture displays the possible occupations of an oxygen vacancy (marked in purple) around the La cation.

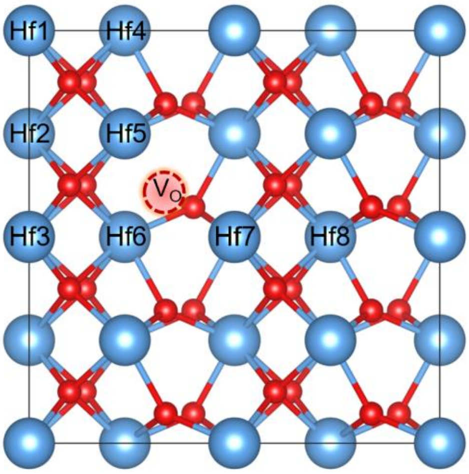

Fig. S14 | Possible configurations of the 2La-Vo system. The oxygen vacancy is fixed in the Vo_3-1 position in accordance with the La-Vo configuration. the Hf1 to Hf8 positions are the postulated sites assuming substituted by La cations.

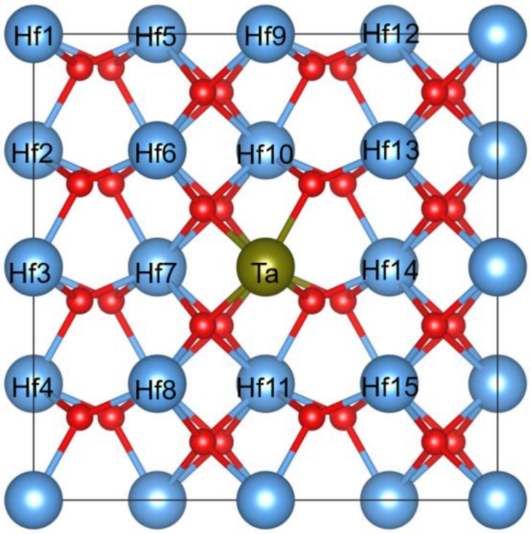

Fig. S15 | Possible configurations of the La-Ta system. The position of a Ta cation is fixed and the Hf1 to Hf15 are different sites for the substituted La cation which was provided to the subsequent thermodynamic calculations.

Table S1 | Energies of different La-Vo configurations.

Configuration	E (meV/f.u.)	Configuration	E (meV/f.u.)
V_O_3-1	0	V_O_4-1	45.10
V_O_3-2	47.30	V_O_4-2	34.93
V_O_3-3	63.93	V_O_4-3	34.45
V_O_3-4	51.84	V_O_4-4	51.93

Table S2 | Energies of different 2La-Vo configurations.

Configuration	E (meV/f.u.)	Configuration	E (meV/f.u.)
La_{Hf1}-V_O-La_{Hf7}	45.17	La_{Hf5}-V_O-La_{Hf7}	42.81
La_{Hf2}-V_O-La_{Hf7}	0	La_{Hf6}-V_O-La_{Hf7}	60.47
La_{Hf3}-V_O-La_{Hf7}	17.27	La_{Hf3}-V_O-La_{Hf8}	36.59
La_{Hf4}-V_O-La_{Hf7}	30.08		

Table S3 | Energies of different La-Ta configurations

Configuration	E (meV/f.u.)	Configuration	E (meV/f.u.)	Configuration	E (meV/f.u.)
Ta-La_{Hf1}	16.18	Ta-La_{Hf6}	6.41	Ta-La_{Hf11}	4.76
Ta-La_{Hf2}	6.05	Ta-La_{Hf7}	1.10	Ta-La_{Hf12}	14.70
Ta-La_{Hf3}	11.48	Ta-La_{Hf8}	0.93	Ta-La_{Hf13}	9.58
Ta-La_{Hf4}	6.51	Ta-La_{Hf9}	12.95	Ta-La_{Hf14}	0
Ta-La_{Hf5}	10.60	Ta-La_{Hf10}	3.60	Ta-La_{Hf15}	5.88

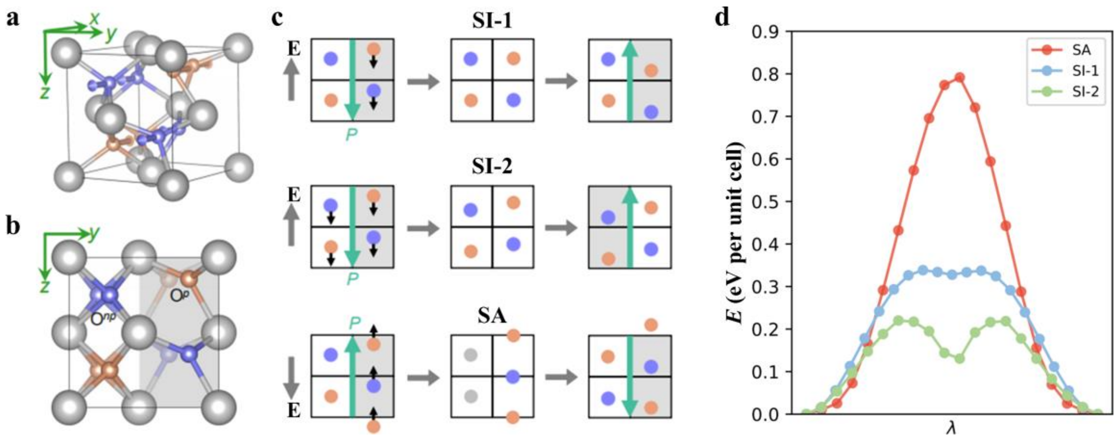

Fig. S16 | Unit-cell-level polarization switching pathways in ferroelectric HfO_2. a X_2^- mode in the unit cell of *Pca2₁* HfO_2. **b** Alternately arranged nonpolar oxygen ions (O^{np}) and polar oxygen ions (O^{p}) in *Pca*2_1 HfO_2. **c** Schematics of shift-inside (SI) and shift-across (SA) switching pathways driven by an external electric field (E). **d** Calculated minimum energy paths for different switching pathways using the DFT-based variable-cell NEB method. [reproduced with permission from ref 9]

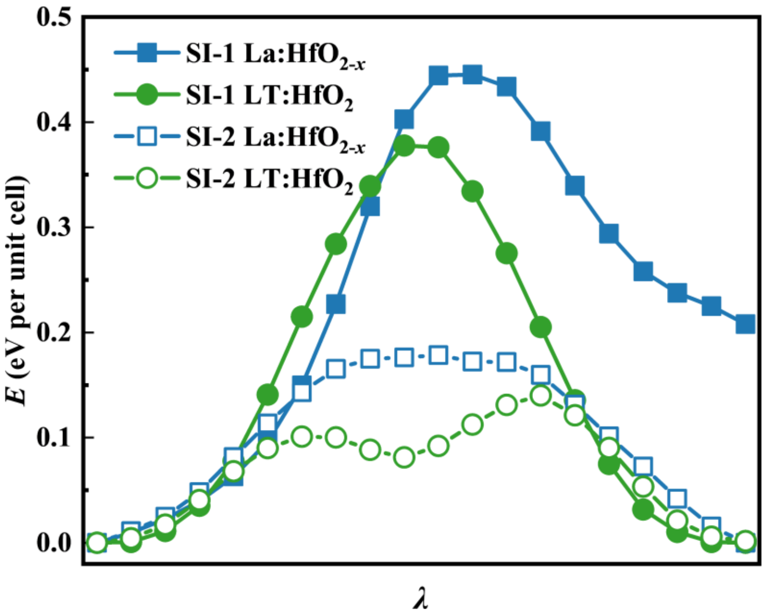

Fig. S17 | Switching barriers for the SI-1 and SI-2 pathways. Switching barriers for the SI-1 and SI-2 pathways in La:$HfO_{2\text{-}x}$ and LT:HfO_2. A 2×2×1 supercell is used.

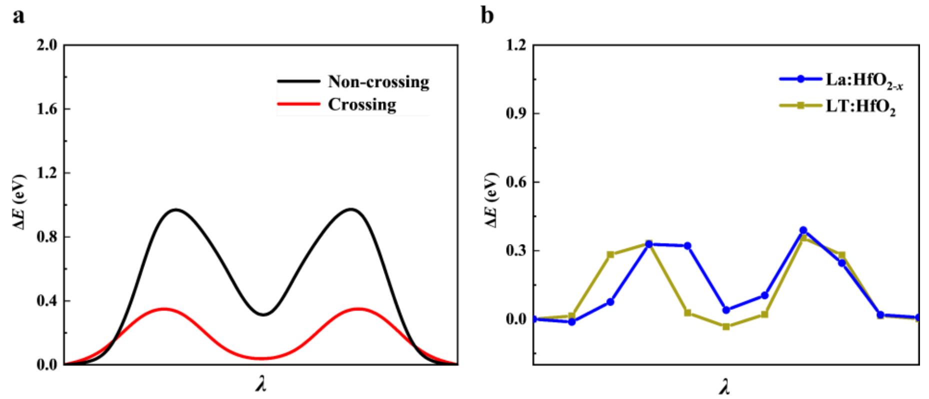

Fig. S18 | Energy barriers for a *Pbca*-type domain wall motion. The barriers of the *Pbca*-type domain wall motion process in **a** pure HfO_2 and **b** La:$HfO_{2\text{-}x}$ and LT:HfO_2.

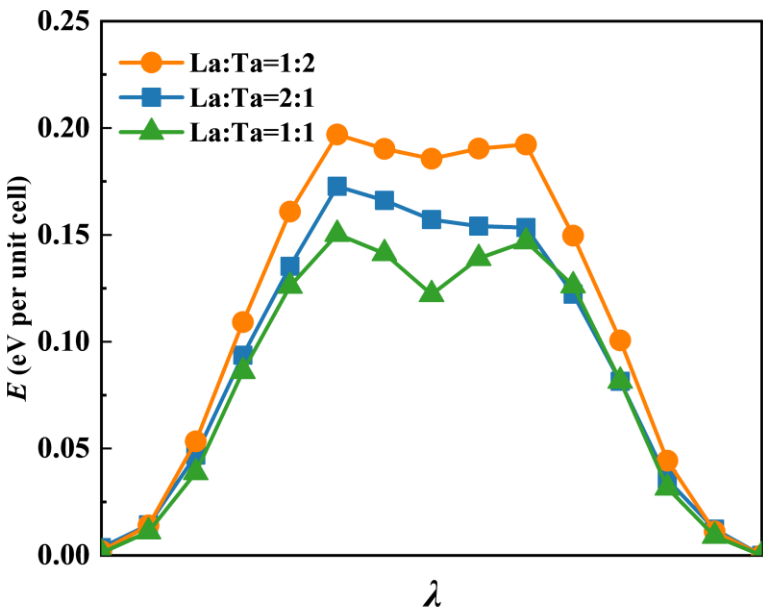

Fig. S19 | DFT minimum energy paths for polarization switching in ferroelectric hafnia with different proportions of La/Ta in a 2×2×2 supercell. The supercell with a La/Ta ratio of 1:1 has the lowest switching energy barrier.

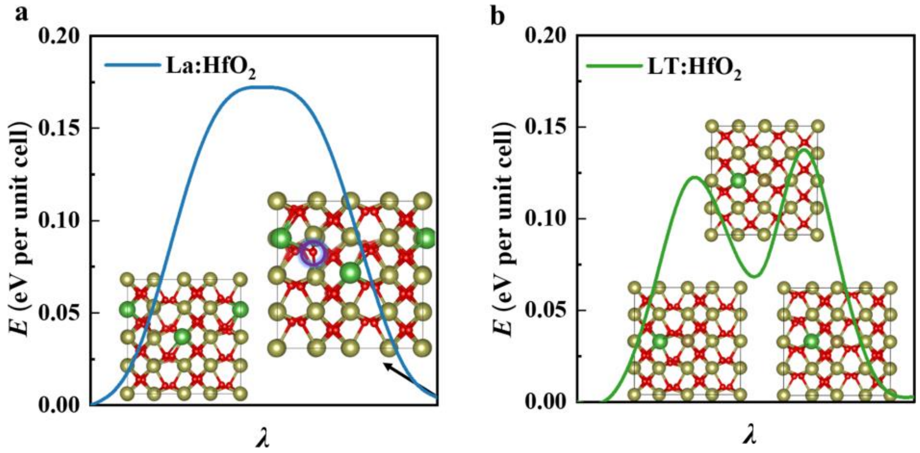

Fig. S20 | Energy barrier for different doping concentrations. Energy barriers of SI-2 switching pathway in **a** La:HfO_2 and **b** LT:HfO_2 using 4×2×2 supercells.
