# Supplementary material for: Enhanced polarization switching characteristics of HfO2 ultrathin films via acceptor-donor co-doping
Source: Nat Commun. 2024 Apr 3;15:2893. doi: 10.1038/s41467-024-47194-8 (PMC10991407; doi:10.1038/s41467-024-47194-8)
Supplement: Supplementary file 3 — Lasing Reporting Summary [file 41467_2024_47194_MOESM3_ESM.pdf]

Corresponding author(s): Shi Liu, Zuhuang Chen

Lasing Reporting Summary

Nature Research wishes to improve the reproducibility of the work that we publish. This form is intended for publication with all accepted papers reporting claims of lasing and provides structure for consistency and transparency in reporting. Some list items might not apply to an individual manuscript, but all fields must be completed for clarity.

For further information on Nature Research policies, including our data availability policy, see Authors & Referees.

• Experimental design

Please check: are the following details reported in the manuscript?

1. Threshold

Plots of device output power versus pump power over a wide range of values indicating a clear threshold

- [ ] Yes
- [x] No

Our research primarily focus on the switching characteristics of ferroelectric HfO2 films. The laser with fixed wavelength and energy serves only for film preparation and parameters such as the threshold are not significant.

2. Linewidth narrowing

Plots of spectral power density for the emission at pump powers below, around, and above the lasing threshold, indicating a clear linewidth narrowing at threshold

- [ ] Yes
- [x] No

Our research primarily focus on the switching characteristics of ferroelectric HfO2. The laser with fixed wavelength and energy serves only for film preparation and parameters such as linewidth narrowing are not significant.

Resolution of the spectrometer used to make spectral measurements

- [ ] Yes
- [x] No

Our research primarily focus on the switching characteristics of ferroelectric HfO2. The laser with fixed wavelength and energy serves only for film preparation and parameters such as linewidth narrowing are not significant.

3. Coherent emission

Measurements of the coherence and/or polarization of the emission

- [x] Yes
- [ ] No

We have provided detailed descriptions of the operating conditions in the Methods-Soft X-ray spectroscopy measurements section.

4. Beam spatial profile

Image and/or measurement of the spatial shape and profile of the emission, showing a well-defined beam above threshold

- [ ] Yes
- [x] No

Our research primarily focus on the switching characteristics of ferroelectric HfO2. The laser with fixed wavelength energy serves only for film preparation and parameters such as beam spatial profile are not significant.

5. Operating conditions

Description of the laser and pumping conditions
Continuous-wave, pulsed, temperature of operation

- [x] Yes
- [ ] No

We have provided detailed descriptions of the operating conditions in the Methods section.

Threshold values provided as density values (e.g. W cm^{-2} or J cm^{-2}) taking into account the area of the device

- [ ] Yes
- [x] No

We did not specify the threshold as a density value as this is not relevant to our study. But we have offered the energy density of the laser fluence in the Methods part.

6. Alternative explanations

Reasoning as to why alternative explanations have been ruled out as responsible for the emission characteristics
e.g. amplified spontaneous, directional scattering; modification of fluorescence spectrum by the cavity

- [ ] Yes
- [x] No

Our research primarily focus on the switching characteristics of ferroelectric HfO2. The laser with fixed wavelength energy serves only for film preparation and parameters such as amplified spontaneous and directional scattering are not significant.

7. Theoretical analysis

Theoretical analysis that ensures that the experimental values measured are realistic and reasonable
e.g. laser threshold, linewidth, cavity gain-loss, efficiency

- [x] Yes
- [ ] No

The switching speed analysis can be found in Fig. 4; structural analysis can be found in Fig. 1 and Figs. S2-S5.

8. Statistics

Number of devices fabricated and tested

- [x] Yes
- [ ] No

We have prepared six 5mm*2.5mm samples. Three of them are patterned with electrodes with different areas to conduct electric test (ferroelectric and dielectric test-Fig. 3, Fig. 4, Fig. S7, Fig. S8, Fig. S10, Fig. S11 and Fig. 12. it should be noted that one sample can be patterned with hundreds of electrodes). One of them is subject to XLD measurement (Fig. 1b), One of them are use to conduct the structure analysis (Fig. 1a, Fig.1c-d, Fig.1f-1g, Fig.S1, Fig. S2, Fig. S3, Fig.S4) and the last one is used for XPS measurement and then is kept as a backup.

Statistical analysis of the device performance and lifetime (time to failure)

☐ Yes
☒ No

Our research primarily focus on the switching characteristics of ferroelectric HfO2 and corresponding samples are prepared as the prototype devices for the characterization of electric performances. There was no statistical analysis on the specific device.
